# scTIDE: Deciphering Critical Transitions Through Cell‐Perturbed Manifold Graphs and Optimal Transport Conditional Flow Matching

**DOI:** 10.1002/advs.76606

**Published:** 2026-07-20

**Authors:** Jiayuan Zhong, Bowen Niu, Yongbo Yu, Shiyang Nie, Xuerong Gu, Pei Chen, Rui Liu

**Affiliations:** ^1^ School of Mathematics Foshan University Foshan China; ^2^ School of Mathematics South China University of Technology Guangzhou China; ^3^ School of Biology and Biological Engineering South China University of Technology Guangzhou China; ^4^ Tianfu Jincheng Laboratory Chengdu China

**Keywords:** critical transition, dynamic network biomarker (DNB), flow matching, manifold, optimal transport

## Abstract

A tipping point marks the threshold or critical state where a biological system shifts from one stable state to another. Deciphering critical transitions and their associated signaling molecules is essential for elucidating complex biological processes and for enabling timely interventions to avert or postpone catastrophic deteriorations. However, existing critical‐state detection methods rely mainly on Euclidean‐space statistics, which may overlook nonlinear dynamical behavior among molecules and distribution‐based molecular patterns, leading to limited robustness and performance in high‐dimensional, sparse, and noisy single‐cell data. In this study, we introduce single‐cell Tipping‐point Identification via Distributional Embedding (scTIDE), a framework that integrates manifold‐based graph representations with optimal‐transport conditional flow matching (OT‐CFM) to capture intrinsic topological structure and identify critical transitions at the individual‐cell level. Specifically, for a given cell, scTIDE quantifies distributional differences between a distribution derived from the reference manifold graph and a perturbed distribution inferred from the cell‐perturbed manifold graph using OT‐CFM, thereby identifying critical stages and key signaling molecules. The reliability and effectiveness of our model are demonstrated through synthetic models and eight distinct single‐cell datasets, where it outperforms existing methods. Moreover, scTIDE reveals possible critical transitions for unseen cells and visualizes the intricate biological progression.

## Graphical abstract

scTIDE identifies single‐cell tipping points by combining manifold‐based graph representations with optimal‐transport conditional flow matching, which preserves intrinsic topology and models distributional dynamics. It supports critical‐transition detection at individual‐cell resolution, prediction of unseen cells, and dimensionality reduction and clustering from PES representation, providing a PES‐based framework for cellular dynamics analysis.

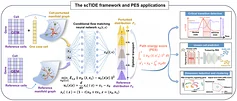

## 1 Introduction

Numerous complex biological processes are characterized by critical transitions, during which systems undergo abrupt and often irreversible changes between distinct dynamical states [1, 2]. Such transitions are widely observed in biological scenarios, particularly in cell fate decisions during differentiation and embryonic development, as well as in the progression of complex diseases [3, 4, 5]. From a systems perspective, biological processes can be regarded as high‐dimensional nonlinear dynamical systems, whose dynamics typically evolve through three states: before‐transition state, critical state at which sudden and catastrophic changes known as critical transition occur, and after‐transition state (Figure 1A) [6]. In contrast to the irreversible after‐transition state, the critical state can restore to the before‐transition state by conducting timely interventions, making it possible to prevent catastrophic events and mitigate negative impacts. However, detecting early‐warning signals of critical transitions and identifying critical states in complex biological systems remain challenging due to high dimensionality, intrinsic cellular heterogeneity, limited sample sizes, sparsity and noise in single‐cell measurements, as well as the nonlinear and dynamic nature of molecular interactions. Moreover, the development from the before‐transition to the critical state is often not accompanied by pronounced differential expression changes, but rather by subtle shifts in the distribution and dynamics of cellular states, making early detection particularly difficult.

**FIGURE 1:**
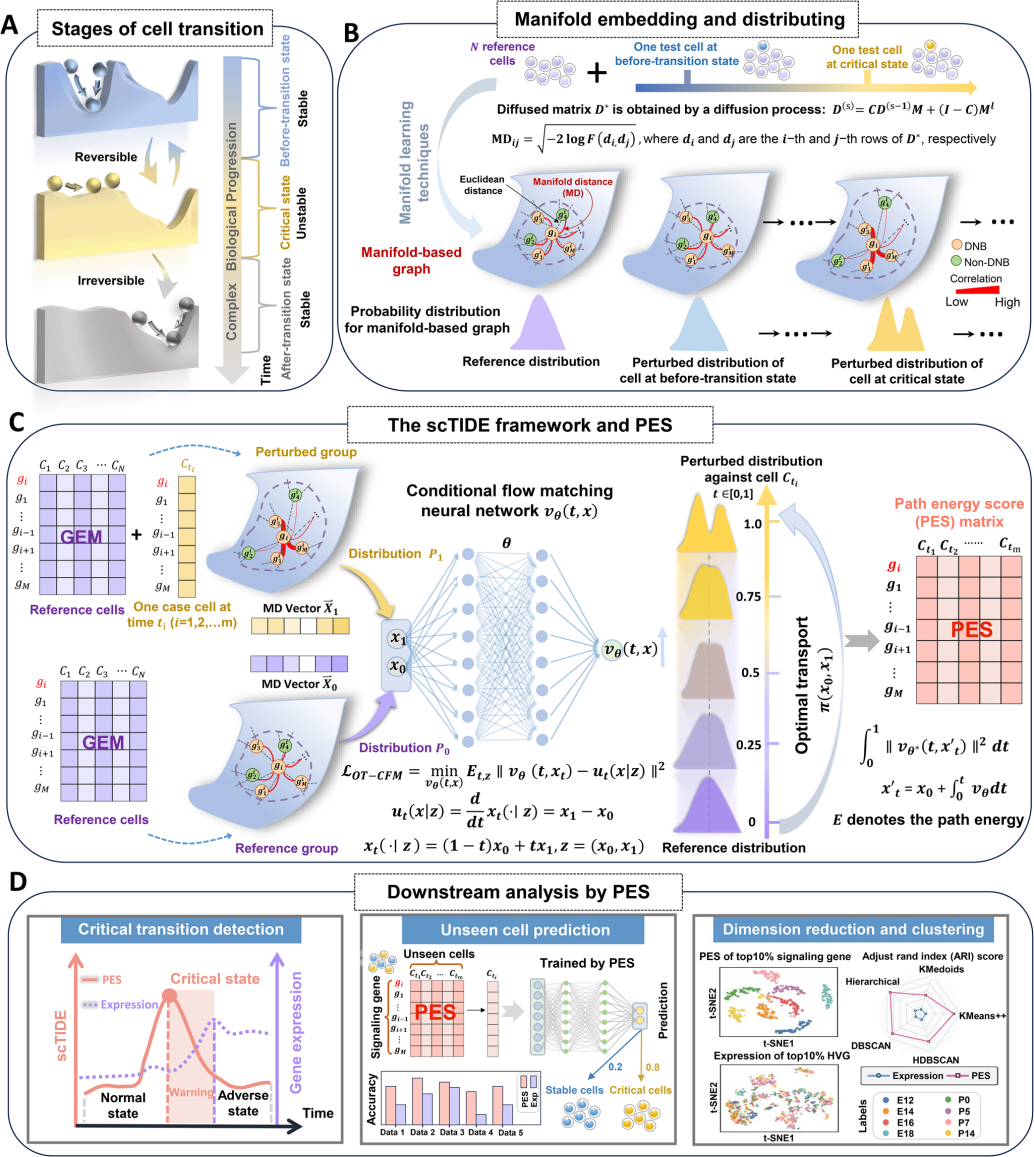
Illustrative schematic of scTIDE for identifying critical transitions. (A) A complex biological system is typically divided into three phases: before‐transition state, critical state where sudden and catastrophic changes known as critical transition occur, and after‐transition state. (B) For each local graph centred on a specific gene, its reference distribution can be derived from the reference manifold‐based graph for the background/reference cells, whereas its perturbed distribution is inferred from the perturbed manifold‐based graph for the mixed cells, which combine an individual case cell with the reference group. (C) Given the reference cells and an individual case cell, scTIDE constructs both reference and perturbed manifold‐based graphs using manifold learning techniques, then quantifies the distributional shifts between the reference and perturbed distributions using optimal‐transport conditional flow matching (OT‐CFM). (D) The critical properties of complex biological systems can be identified by scTIDE, with a significant increase in its path energy score (PES) indicating the occurrence of critical transitions. Additionally, scTIDE also facilitates downstream analyses, including critical‐state prediction for unseen cells as well as PES‐based dimensionality reduction and clustering.

Diverse strategies and techniques have been proposed to identify critical transitions. For instance, a cohesive mathematical framework that incorporates bifurcation theory, fast–slow dynamics, and stochastic processes has been developed, providing fundamental insights into the mechanisms and predictability of critical transitions in complex systems [7]. Computational strategies founded on the non‐equilibrium system's flux theory have been designed to assess different characteristics of state transitions within the system [8, 9]. Despite having a solid theoretical foundation, these methods face challenges in large‐scale, high‐dimensional biological datasets due to their high computational complexity. More recently, the dynamic network biomarkers (DNB) framework, rooted in the theory of critical slowing down, has been proposed to capture dynamic changes in molecular behavior and detect key transitions during complex biological processes [10, 11]. Building on this framework, several methods, including sample‐perturbed Gaussian graphical model (sPGGM) [3], Gaussian graphical optimal transport (GGOT) [4], personalized edge‐network biomarkers (PDENB) [12], module‐based dynamic network biomarkers (M‐DNB) [13], single‐sample landscape entropy (SLE) [14], and directed network flow entropy (DNFE) [15], have been developed to identify critical transitions in complex biological systems. Despite their utility, these approaches primarily rely on Euclidean‐space statistics and thus mainly capture coordinate‐wise statistical variations. As a result, they often overlook nonlinear dynamical behaviors among molecules and distributional changes of molecular patterns when applied to high‐dimensional, sparse, and noisy single‐cell datasets. First, in high‐dimensional expression spaces, the distance concentration effect associated with the “curse of dimensionality” reduces the discriminative power of Euclidean distance metrics [16, 17], thereby weakening sensitivity to topology‐level changes induced by molecular regulatory reorganization [18]. Second, Euclidean statistics are highly susceptible to the inherent sparsity of single‐cell data, which restricts their ability to resolve biologically meaningful dynamics in high‐dimensional systems [19, 20, 21]. More importantly, critical transitions are governed by nonlinear dynamical interactions among molecules, whereas Euclidean distance, as a coordinate‐wise linear aggregation metric, is not well suited to characterize dynamical changes in nonlinear dependency structures near critical states [2, 22].

In contrast to conventional Euclidean distances, manifold‐based metrics provide a complementary perspective by explicitly accounting for the intrinsic geometry of data embedded in nonlinear high‐dimensional spaces [23]. By estimating geodesic distances on curved manifolds, such approaches offer a principled way to uncover nonlinear relationships and hidden patterns in high‐dimensional, sparse single‐cell data. In particular, diffusion‐based manifold distances leverage random walks on graphs to achieve an effective balance between local neighborhood information and global topological organization, thereby enabling the capture of topology‐level changes overwhelmed by high‐dimensional effects [24]. Moreover, from a dynamical systems viewpoint, as a system approaches a critical state, its dynamics progressively collapse onto the center manifold and become governed by a subset of coordinately fluctuating dominant variables (DNBs), leading to reduced resilience to perturbations and amplified fluctuations along specific low‐dimensional modes [7, 23]. A diffusion‐based manifold can provide a practical approximation to this underlying manifold by capturing the intrinsic geometry and dynamical evolution of the data, thereby enabling a more informative characterization of nonlinear dynamics near tipping points.

Recently, a wide range of optimal transport‐guided modeling approaches, including Waddington‐OT [25], TIGON [26], TrajectoryNet [27], and MIOFlow [28], have been developed to couple temporal distributions and infer continuous dynamic cellular trajectories by calculating the minimal transition cost between cell states. Nevertheless, conventional optimal transport strategies typically require substantial computational resources due to their iterative optimization steps, restricting their ability to handle large‐scale, high‐dimensional datasets [29]. Notably, flow matching [30, 31] serves as a simulation‐free method that efficiently trains the velocity field by constructing conditional probability paths without the need for trajectory simulation, which provides a powerful means to alleviate computational burdens. Building upon this foundation, further advancements have been made by integrating optimal transport techniques [32, 33], considering manifold structures [34], and estimating the Schrödinger bridge using flow and score matching [35], all of which have been utilized in modelling single‐cell dynamics. Therefore, drawing from these advances, incorporating manifold‐based distance, optimal transport theory, and flow matching into the framework of critical‐state detection may offer an insightful avenue for capturing distributional and dynamical changes associated with critical transitions.

In this work, we introduce a novel framework of scTIDE, which combines manifold‐based graphs with the optimal‐transport conditional flow matching (OT‐CFM) to decipher critical transitions and identify signaling molecules in the dynamical evolution of complex biological systems at the individual‐cell level. Specifically, our proposed scTIDE constructs a reference manifold‐based graph for background/reference cells and a perturbed manifold‐based graph for mixed cells (comprising an individual case cell and the reference group) using a manifold learning technique (Figure 1B). Subsequently, for each local graph centred on a specific gene, scTIDE captures distributional shifts between its reference distribution (derived from the reference manifold‐based graph) and its perturbed distribution (inferred from the perturbed manifold‐based graph) through the OT‐CFM framework (Figure 1C), thereby quantifying the minimal “effort” required for transitions from before‐transition to critical states. The critical properties of complex biological systems can be revealed by scTIDE, with notable increases in its path energy score (PES) serving as early warning signals for critical transitions. The proposed scTIDE exhibits key methodological advances over existing critical‐transition detection methods as follows. First, compared with existing methods that mainly rely on Euclidean‐space statistics or coordinate‐wise molecular variations, scTIDE constructs cell‐perturbed manifold graphs to capture nonlinear, topology‐level reorganization of molecular interactions in high‐dimensional single‐cell data, enabling the detection of subtle geometric changes that are not effectively characterized by conventional approaches. Second, scTIDE introduces an optimal‐transport conditional flow matching framework to quantify distributional shifts in local manifold graphs, which improves robustness to noisy single‐cell data and enables more stable and accurate detection of early‐warning signals. Third, beyond identifying critical transitions at the individual‐cell level, scTIDE further supports critical‐state prediction in unseen cells, as well as dimensionality reduction and clustering from the perspective of PES representation (Figure 1D), providing a PES‐based framework for the analysis of cellular dynamics with improved performance over conventional expression‐based approaches.

To validate the effectiveness and robustness of scTIDE, we applied it to both simulated and eight distinct real‐world single‐cell datasets, covering disease progression‐related contexts such as mouse myocardial infarction, cerebellar tumor cells progression, and neuroendocrine transitions in small cell lung cancer, as well as developmental processes including murine pancreatic development, liver development, human embryonic stem cell differentiation, induced pluripotent stem cell differentiation, and radial progenitor proliferation. The results demonstrate that our scTIDE exhibits strong predictive power for critical transitions in various biological processes and uncovers signaling molecules associated with these pivotal points. Furthermore, in comparison to other existing critical‐state detection methods, scTIDE exhibits enhanced noise resilience under varying noise conditions and shows better performance in detecting critical signals across diverse single‐cell datasets. Additionally, the functional implications of the signaling molecules further support the effectiveness of scTIDE. By combining manifold‐based graph and OT‐CFM, scTIDE provides a novel framework for deciphering critical transitions and uncovering signaling molecules at the individual‐cell level, shedding light on the dynamic behavior and bifurcation analysis of complex biological systems.

## 2 Results

### 2.1 A Manifold‐Based View of Tri‐State Dynamics in Biological Processes

Complex biological processes, such as disease progression and embryonic development, often undergo sudden shifts in temporal dynamics and can be considered evolving nonlinear dynamical systems, where critical transitions mark a qualitative change at a bifurcation juncture or tipping point [35, 36]. Such a dynamic progression is typically modeled as three distinct states (Figure 1A): (1) a stable before‐transition state with strong robustness; (2) an unstable, low‐resilience critical state, known as a critical transition; and (3) another stable after‐transition state with high resilience. Our recently proposed theory of dynamical network biomarkers (DNB) [11, 37] suggests that a set of molecules, defined as the DNB, exhibits strong correlations and synchronized fluctuations in their behavior, when the system nears the tipping point (see Section S2 for details).

From a dynamic perspective, the critical state corresponds to a system approaching a bifurcation, in which stability progressively decreases. Biological progression can be described by a stochastic differential equation *d**X *** =  *G*(***X***; ***P***)*dt* + σ*d**W**_t_*, where ***X*** denotes the system state, ***P*** represents slowly varying control parameters (e.g., environmental factors, genetic mutations, or lifestyle influences), and ***W**_t_* captures stochastic perturbations. As the system parameters evolve toward a bifurcation point, a previously stable attractor loses stability as the dominant eigenvalue of the Jacobian matrix of *G* approaches zero. Consequently, the system dynamics contract onto a low‐dimensional center manifold $M_{c}$. In the presence of stochastic perturbations, fluctuations along stable directions decrease rapidly, whereas variability along the center‐manifold directions is preserved and increases.

In single‐cell experiments, cellular states are observed as stochastic snapshots of the underlying dynamics. Near a critical transition, contraction onto the center manifold constrains cellular variability to a low‐dimensional subset of the high‐dimensional expression space. Statistically, this constrained variability manifests as a curved, low‐dimensional geometric structure embedded in the high‐dimensional space, giving rise to an intrinsic data manifold. This data manifold can therefore roughly serve as a statistical and geometric approximation of the underlying center‐manifold dynamics near critical transitions [38, 39].

Importantly, the properties of DNBs indicate that critical transitions are characterized by dynamic reorganization at the network level. By transforming molecular expression patterns into a diffusion‐informed manifold‐based graph representation that preserves the intrinsic system geometry, nonlinear molecular dynamics associated with critical transitions can be more effectively characterized [18, 38]. Distributional embedding integrates local neighborhood structure with global network organization, enabling the characterization of dynamic changes in manifold‐based molecular interactions and the deciphering of evolving system‐level dynamical patterns [40]. As the system approaches the critical state, correlations among DNB molecules are markedly strengthened, leading to reduced manifold distances within the DNB module, while correlations between DNB and non‐DNB molecules weaken, resulting in increased separation in manifold space (Figure 1B). Thus, distributional embedding may provide a principled framework for capturing nonlinear dynamic shifts in manifold‐based molecular organization and deciphering critical transitions in complex biological systems.

### 2.2 The Overview of scTIDE

scTIDE adopts a diffusion‐based manifold learning strategy to construct intrinsic geometric representations of high‐dimensional molecular expression profiles (Figure 1B). By constructing a k‐nearest‐neighbor graph and applying a personalized PageRank–based diffusion process (as defined in Equation (1)), scTIDE embeds molecular expression profiles into a manifold‐based graph representation.

(1) $$D^{\left(s\right)}=CD^{\left(s-1\right)}M+\left(I-C\right)M^{l}$$

which converges to a stationary diffusion state D* as *s* → ∞, and *C* is the learnable damping factor (see details in “Diffusion‐informed manifold‐based graphs” section of Methods and Materials). Moreover, based on the diffused representation D*, the manifold geodesic distance MD_*ij*_ on the manifold M between the *i*‐th and the *j*‐th nodes/genes is defined using kernel‐based similarity measures as follows.

(2) $$MD_{ij}=\sqrt{-2\log F\left(d_{i},d_{j}\right)}\;,$$

where *F* denotes the Bhattacharyya kernel applied to diffusion‐based probability vectors, which is formulated as the inner product $F\left(d_{i},d_{j}\right)=<\sqrt{d_{i}},\sqrt{d_{j}}>$ (with the vectors ***d***_***i***_ and ***d***_***j***_ representing the *i*‐th and *j*‐th rows of D*,  respectively) [41, 42]. Such a manifold distance provides a geometry‐aware metric for quantifying molecular relationships beyond Euclidean proximity. Specifically, Euclidean‐distance‐based statistics are susceptible to the curse of dimensionality and distance concentration effects, which limit their ability to capture topology‐level changes in high‐dimensional expression spaces. Moreover, Euclidean representations are sensitive to the intrinsic sparsity and noise of single‐cell data and may fail to capture biologically meaningful nonlinear relationships among molecules. In contrast, manifold‐based graphs preserve the intrinsic geometry and topological organization of the data, providing a more informative representation of molecular interactions. In particular, diffusion‐based manifold representations integrate both local neighborhood information and global topological structure, enabling the detection of topology‐level changes that are often obscured in high‐dimensional, noise‐prone single‐cell data near critical transitions. Within this manifold representation, scTIDE extracts gene‐centered local manifold‐based graphs, which serve as fundamental units for characterizing system dynamics. For each gene, probability distributions are constructed from normalized manifold distances to its local neighbors under reference (stable) and perturbed (potentially critical) conditions, allowing molecular interactions to be represented as distributions rather than isolated point estimates. This formulation naturally facilitates scTIDE to focus on collective structural changes, in line with the DNB hypothesis emphasizing coordinated molecular behavior near critical transitions [11, 37].

To quantify the dynamic changes in distributional embedding representations derived from manifold‐based graphs across different system states, scTIDE integrates optimal transport theory with conditional flow matching (Figure 1C). Given reference and perturbed distributions *p*_0_ and *p*_1_ defined on the manifold in Equation (2), the optimal transport problem seeks a coupling π ∈ Γ(*p*_0_,*p*_1_) that minimizes the transport cost

(3) $$\min_{\pi \in \Gamma \left(p_{0},p_{1}\right)}\int_{M\times M}{\left\|x_{1}-x_{0}\right\|}^{2}\;d\pi \left(x_{0},x_{1}\right)$$

where Γ(*p*_0_,*p*_1_) denotes the set of all joint distributions π such that the marginal distributions of π correspond to *p*_0_ and *p*_1_​ (i.e., π (*x*_0_,·) = *p*_0_(*x*_0_) and π (·,*x*_1_) = *p*_1_(*x*_1_)), with *x*_0_ and *x*_1 _ representing data points sampled from the source distribution *p*_0_ and the target distribution *p*_1_, respectively. Rather than explicitly solving for π, scTIDE parameterizes the transport dynamics via a time‐dependent velocity field *v*_θ_(*t*, *x*): $\left[0,1\right]\times M\to T_{M}$, which is trained to approximate a conditional velocity by minimizing the following loss:

(4) $$\;L_{OT-CFM}=\min_{v_{\theta }\left(t,x\right)}\;E_{t,\;z}∥v_{\theta }\left(t,x_{t}\right)-u_{t}\left(x|z\right){∥}^{2},\;$$

with

(5) $$u_{t}\;\left(x|z\right)=\frac{d}{dt}\;x_{t}\left(\cdot |z\right)=x_{1}\;-x_{0}.$$

Here, *z*  = (*x*_0_, *x*_1_)  denotes the source‐target pair, and *x_t_* (· |*z*)  = (1 − *t*) *x*_0_ + *tx*_1_ ​ represents a time‐dependent point along the transport trajectory from *x*_0_ to *x*_1_. The loss function defined in Equation 4 describes how the neural network *v*_θ_(*t*, *x_t_*) regresses the real velocity *u_t_* (*x*) based solely on a sampled point *z*. Actually, it is not practicable to learn *v*_θ_(*t*, *x*) to approximate a real velocity *u_t_* (*x*) by minimizing $\min_{v_{\theta }\left(t,x\right)}E_{t,\;x}∥v_{\theta }\left(t,x\right)-u_{t}\left(x\right){∥}^{2}$, due to the inherent unknown nature of *u_t_* (*x*). Since the conditional loss defined by Equation (4) is gradient‐equivalent to the flow matching loss $\min_{v_{\theta }\left(t,x\right)}E_{t,\;x}∥v_{\theta }\left(t,x\right)-u_{t}\left(x\right){∥}^{2}$ [43, 44], the posterior probability average or optimal parameter network $v_{\theta^{*}}\left(t,x\right)$ can be employed to closely approximate the real velocity *u_t_* (*x*). Building on the resulting optimal velocity field $v_{\theta^{*}}\left(t,x\right)$, scTIDE defines a path energy score (PES) as follows:

(6)

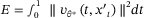

which quantifies the minimal dynamical effort required to transport the system from the reference distribution toward the perturbed distribution. As the system approaches a tipping point, the divergence between reference and perturbed distributions increases sharply, resulting in a pronounced rise in PES that serves as a quantitative and geometry‐aware indicator of critical transitions.

Overall, scTIDE establishes a unified theoretical and computational framework that bridges nonlinear dynamics, diffusion‐based manifold geometry, and optimal transport theory. By leveraging intrinsic geometric representations in the form of nonlinear manifold graphs and transport‐driven distributional dynamics, scTIDE enables characterization of system‐level instability, robust detection of critical transitions, and identification of key signaling molecules driving critical state changes at the individual‐cell level. This framework not only enhances sensitivity and robustness in high‐dimensional, sparse single‐cell data but also provides new insights into the nonlinear dynamical evolution of complex biological systems.

The proposed scTIDE mainly supports the following PES‐based downstream analyses (Figure 1D), with the detailed descriptions provided in the Methods section.

1. characterization of complex biological progression and identification of critical transitions at the individual‐cell level;
2. prediction of the likelihood of critical states in unseen cells using a deep learning classifier trained on PES of signaling genes;
3. transformation of the sparse single‐cell gene expression profiles into non‐sparse PES representations, enabling PES‐based dimensionality reduction and clustering.

### 2.3 Validation of the scTIDE Through Numerical Simulations

To test the effectiveness and stability of the scTIDE model, we employed an 18‐node artificial network (Figure 2A) to demonstrate the identification of early‐warning signals as the system approaches a tipping point. This type of regulatory network is described by a stochastic differential system using Michaelis–Menten or Hill equations, widely applied in the analysis of gene regulation in biological processes, such as transcription, translation, and other intricate molecular interactions [45, 46]. The network dynamics are controlled by the equation parameter *p*, with *p*  =  0 signifying a critical threshold identified as the tipping point (see Section S3 for more details). Nodes 1 to 7 in the regulatory network, acting as dominant variables (DNBs) or signaling molecules, are directly governed by the parameter *p*, whereas the remaining nodes are not impacted by *p* and serve as non‐relevant molecules. A parameter ranging from ‐0.4 to 0.2 was utilized to generate simulated datasets, demonstrating how well the scTIDE identifies the critical transition near the bifurcation point.

**FIGURE 2:**
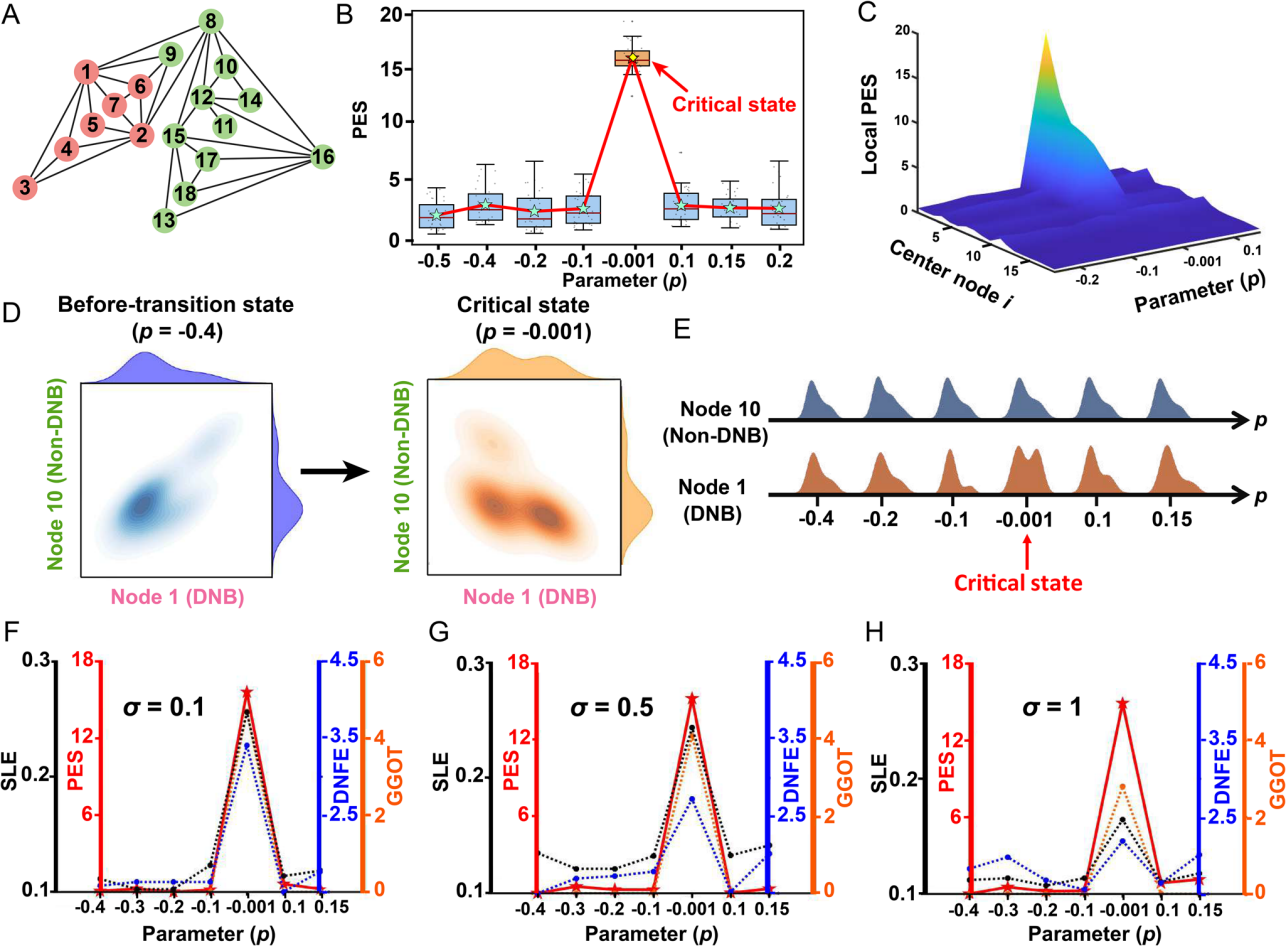
Evaluation of scTIDE's performance through a simulation experiment. (A) A numerical experiment is conducted with an 18‐node graph, which is designed by a gene‐modulated network to model the relationships among the nodes. (B) The curve of path energy score derived from scTIDE exhibits a steep increase at a particular parameter (*p*  =  0), signaling an imminent critical transition. (C) The landscape of path energy score demonstrates a steep increase in the scores of specific local networks, centered around signaling molecules or DNBs (1,  2,  3,  …,  7), as the system nears the tipping point. (D) The variations in the joint distribution of nodes 1 (DNB) and 10 (non‐DNB) between the before‐transition state (*p*  =   −0.4) and the critical state (*p*  =   −0.001). (E) A progression comparison of the distribution for local manifold‐based graphs, centered separately on nodes 1 (DNB) and 10 (non‐DNB), at various parameter values (*p*). (F–H) Evaluation of scTIDE's stability in comparison to other critical state detection algorithms under different noise intensities.

Figure 2B shows the evolving changes in the path energy score (PES) *E*_*t* _ (defined by Equation (12)), where a notable surge at a specific parameter (*p*  =  0) signals the impending of a critical state. To further capture the dynamics of DNBs or signaling molecules during progression, we present the evolution of local PES for different local networks (Figure 2C). When the system is distant from the tipping point, the PES across all local networks stay consistently low and stable. However, as the system draws closer to the critical state, a marked increase in the local PES is observed in specific local networks centered on DNB nodes (1, 2, 3, ···, 7). Furthermore, as shown in Figure 2D,E, distribution transport process is depicted to effectively highlight the differences between the before‐transition and critical states. As the system gets closer to the critical state, the distribution for local manifold‐based graphs centered on DNBs gradually diverges and exhibits increased fluctuations, indicating a significant rise in the geodesic distances on the manifold among DNBs. To assess model robustness, we compared scTIDE with other critical‐state detection methods under varying noise levels (Figure 2F–H). As the noise intensity increases, scTIDE demonstrates superior performance in consistently providing reliable indicators of critical transitions, validating its enhanced robustness and performance, particularly in the presence of elevated noise levels. Overall, the simulation experiment demonstrates that scTIDE is both reliable and accurate in deciphering critical transitions and pinpointing key signaling molecules.

### 2.4 Identification of Critical Transitions for Various Biological Processes

In this study, we demonstrate the capabilities of scTIDE by applying it to eight distinct single‐cell datasets: encompassing disease evolution‐related contexts such as mouse myocardial infarction (MMI) progression [47], cerebellar tumor cells (CTC) progression in childhood [48], neuroendocrine transitions in small cell lung cancer (SCLC) [49], as well as developmental processes including murine pancreatic (MP) development [50], liver development [51], human embryonic stem cell (hESC) differentiation [52], induced pluripotent stem cell (iPSC) differentiation [53], and radial progenitor (RD) proliferation [54]. The PES for each individual cell was calculated according to the procedure described in the “Deciphering Critical Transitions via scTIDE” section of the Methods and Materials. Moreover, by averaging the PES of cells at specific time points, it can detect potential key transition stages. These results demonstrate the effectiveness of scTIDE in identifying critical transitions across various biological processes. The time points associated with key reported biological observations and their corresponding critical states identified by scTIDE across different datasets are provided in Table S1. The source code of the model is available for free at https://github.com/Terry‐NIU/scTIDE.

In disease evolution‐related contexts of single‐cell datasets, for MMI progression data, two critical transitions are identified by a sudden increase in the PES (top panel of Figure 3A): the first critical state occurs at Day 3 (*P*_1_ =  2.33E − 3 and CSD  =  0.53) and the second at Day 14 (*P*_2_ =  1.31E − 18 and CSD  =  1.18). These signals align with experimental observations that the high prevalence of cardiac rupture in hearts occurs during the early proliferative phase (Day 3–7), while during the maturation phase (Day 14–28), fibroblasts and endothelial cells dominate over immune cells, marking a pivotal time window of pathological remodeling that serves as a predictor of disease outcomes [47]. Similarly, for CTC progression data, as shown in the top panel of Figure 3B, two notable increases in the PES are observed at E16 (*P*_1_ =  9.88E − 8 and CSD  =  0.62) and P0 (*P*_2_ =  1.33E − 21 and CSD =  1.24), signaling that Purkinje cells and GABA interneurons in the GABAergic lineage begin to appear during the embryonic stages (E16‐E18), while the glial lineage emerges after P0, with astrocytes forming throughout postnatal stages [48]. When applied to SCLC neuroendocrine transition data, there is a significant increase in the PES at Day 11 (top panel of Figure 3C) (*P*  =  2.07E − 14, CSD = 1.21), after which high expression levels of neuroendocrine markers are largely absent and replaced by expression upregulation of non‐neuroendocrine markers, indicating a transition from a neuroendocrine to a non‐neuroendocrine tumor phenotype [49].

**FIGURE 3:**
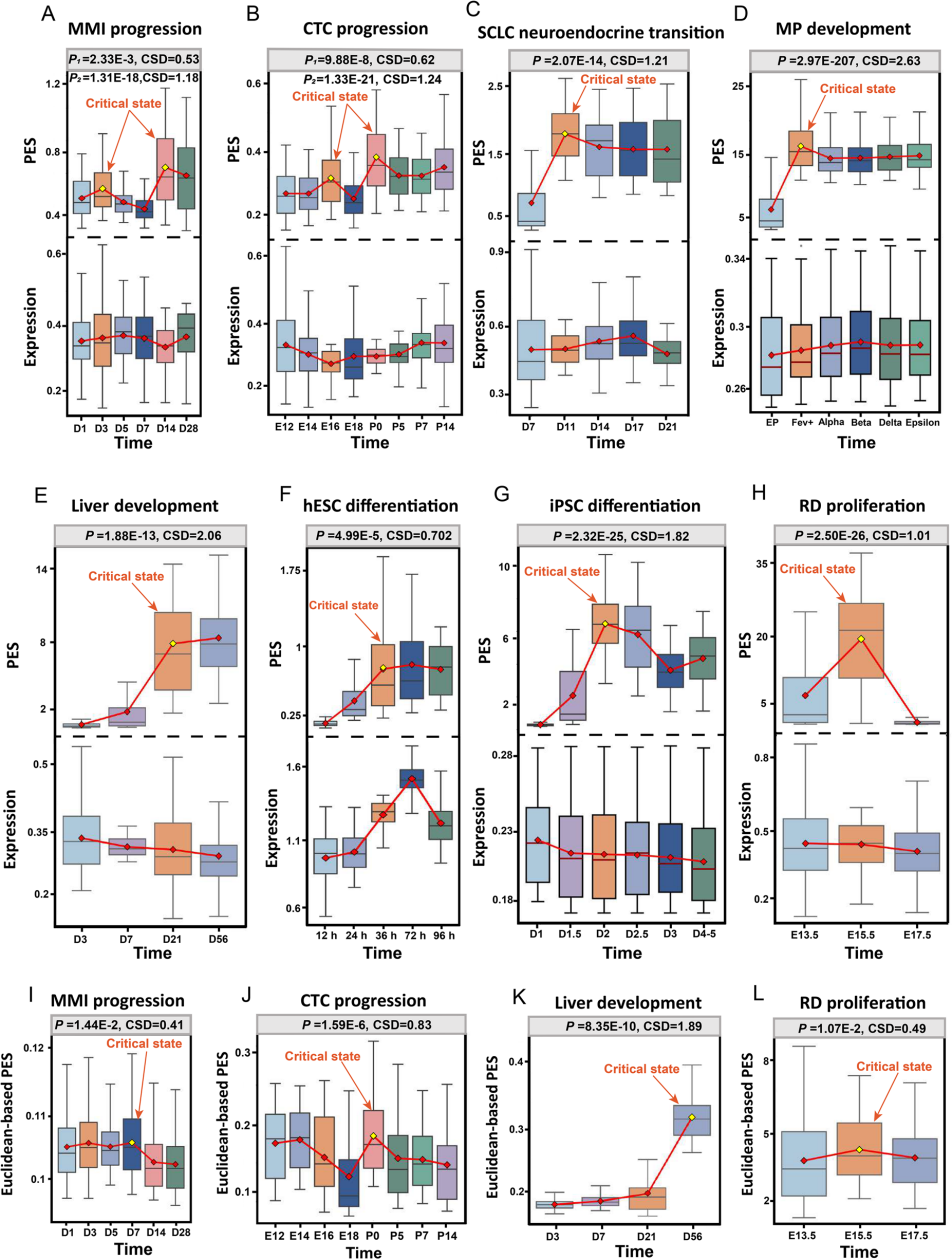
Identification of critical transitions in various biological processes. A comparison of dynamic behavior is performed between scTIDE and highly variable gene (HVG) expression across eight single‐cell datasets: (A) MMI progression, (B) CTC progression, (C) SCLC neuroendocrine transition, (D) MP development, (E) liver development, (F) hESC differentiation, (G) iPSC differentiation, and (H) RD proliferation. Additionally, the performance of the Euclidean‐based path energy score (PES) is evaluated on four of these datasets: (I) MMI progression, (J) CTC progression, (K) liver development, and (L) RD proliferation.

In terms of single‐cell datasets related to developmental processes, the PES of MP development data shows a significant increase (*P*  =  2.97E − 207, CSD  = 2.63) in the Fev+ cluster (top panel of Figure 3D), followed by cells traversing trajectories toward alpha, beta, epsilon, and delta cell fates [50]. For liver development data, it is seen from the top panel of Figure 3E that the PES exhibits a significant increase from Day 7 to 21 (*P*  =  1.88E − 13, CSD = 2.06), suggesting that the critical transitional state of hepatocytes occurs at Day 21. This finding aligns with the original experimental observation that intermediate‐state cells ultimately differentiate into mature hepatocytes at Day 56 [51]. When applied to hESC differentiation data, a significant shift in (*P*  =  4.99E − 5, CSD =  0.702) the PES is observed at 36 h (top panel of Figure 3F), signaling an impending cell fate transition. Indeed, the differentiation trajectory toward a definitive endoderm (DE) fate commitment after 36 h has been recorded, with DE induction occurring at 72 h [52]. In iPSC differentiation data, the PES shown in the top panel of Figure 3G exhibits an upward trend from Day 1.5 to 2 (*P*  =  2.32E − 25, CSD  =  1.82), after which the primitive streak (PS)‐like progenitors transition into either mesodermal or endodermal states, as reflected by lineage‐specific transcripts [53]. For RD proliferation data, a notable difference in the PES occurs at E15.5 (*P*  =  2.50E − 26, CSD =  1.01), signaling imminent critical transitions into a non‐proliferative state after E15.5 (top panel of Figure 3H) [54].

To compare scTIDE with expression‐based signals, we computed a summary statistic based on the top 10% highly variable genes (HVGs) for each individual cell. However, as shown in the bottom panels of Figure 3A–H, the expression patterns of HVGs do not effectively capture dynamic shifts and critical transitions as effectively as PES, either in terms of signal strength or interpretability. Additionally, a comparative analysis was conducted between scTIDE and an alternative variant in which Euclidean‐space graphs were used in place of manifold‐based graphs while keeping the remaining pipeline unchanged. The signal provided by scTIDE is notably more significant than that of the Euclidean‐based PES variant (Figure 3I–L). Therefore, in this study, we utilized manifold‐based graphs to generate the PES, demonstrating its powerful ability in analyzing the dynamic changes of complex biological processes. Moreover, in contrast to the KL‐divergence‐based variant, the proposed OT‐CFM‐based scTIDE yields stronger critical signals and achieves more accurate detection of critical‐transition states (Figure S1), demonstrating that the OT‐CFM module enhances warning‐signal capability by explicitly modeling the transport process between distributions. Besides, compared to five existing critical state detection methods [3, 4, 13, 14, 15] (Table S2), our scTIDE shows enhanced performance in identifying critical transitions throughout the progression of complex biological systems (Table 1).

**TABLE 1:** Performance comparison among different critical transition detection methods.

	Metrics	scTIDE	SLE	DNFE	GGOT	sPGGM	M‐DNB
**SCLC progression**	*P*‐value	**2.07E‐14** **(Day 11)**	None	None	None	3.63E‐4 (Day 11)	7.34E‐4 (Day 14)
	CSD	**1.21** **(Day 11)**	None	None	None	0.48 (Day 11)	3.70 (Day 14)
**MMI progression**	*P*‐value	**2.33E‐3** **(Day 3)** **1.31E‐18** **(Day 14)**	2.24E‐4 (Day 5) 1.06E‐4 (Day14)	3.50E‐7 (Day 5)	1.455E‐2 (Day 3) 1.676E‐2 (Day 14)	1.05E‐2 (Day 3)	4.52E‐2 (Day 3)
	CSD	**0.53** **(Day 3)** **1.18** **(Day 14)**	0.44 (Day 5) 0.38 (Day 14)	1.29 (Day 5)	0.31 (Day 3) 0.91 (Day 14)	0.38 (Day 3)	0.48 (Day 3)
**RD proliferation**	*P* ‐value	**2.50E‐26** **(E15.5)**	None	1.79E‐7 (E15.5)	None	8.69E‐3 (E15.5)	None
	CSD	**1.01** **(E15.5)**	None	0.44 (E15.5)	None	0.18 (E15.5)	None
**Liver development**	*P* ‐value	**1.88E‐13** **(Day 21)**	5.56E‐3 (Day 7)	1.41E‐2 (Day 7)	3.67E‐4 (Day 21)	1.63E‐15 (Day 56)	None
	CSD	**2.06** **(Day 21)**	0.38 (Day 7)	0.32 (Day 7)	1.24 (Day 21)	1.09 (Day 56)	None
**CTC progression**	*P* ‐value	**9.88E‐8** **(E16)** **1.33E‐21** **(P0)**	6.87E‐4 (P0)	2.10E‐9 (P0)	1.75E‐2 (E16) 1.76E‐2 (P0)	6.90E‐7 (E14)	2.85E‐6 (E16)
	CSD	0.62 (E16) **1.24** **(P0)**	0.32 (P0)	0.68 (P0)	0.26 (E16) 0.24 (P0)	0.71 (E14)	**1.35** **(E16)**
**iPSC differentiation**	*P* ‐value	**2.32E‐25** **(Day 2)**	4.25E‐3 (Day 1.5)	2.91E‐12 (Day 3)	2.12E‐22 (Day 1.5)	4.91E‐9 (Day 2.5)	None
	CSD	1.82 (Day 2)	0.36 (Day 1.5)	0.82 (Day3)	**2.04** **(Day1.5)**	0.67 (Day 2.5)	None
**MP development**	p‐value	**2.97E‐207** **(Fev+)**	6.79E‐7 (Delta)	4.20E‐2 (Fev+)	3.31E‐204 (Fev+)	6.23E‐15 (Beta)	7.96E‐3 (Fev+)
	CSD	2.63 (Fev+)	0.74 (Delta)	0.92 (Fev+)	**2.75** **(Fev+)**	0.49 (Beta)	2.63 (Fev+)

### 2.5 Analysis of PES‐Based Dimension Reduction and Clustering

In our study, the top 10% of genes with the highest local PES are classified as signaling genes or DNBs, which are notably responsive to critical transitions preceding catastrophic events. To give a comprehensive view of PES changes, the PES landscape for four single‐cell datasets (MMI progression, CTC progression, SCLC neuroendocrine transition, and liver development) is shown in Figure 4A, where the PES of signaling genes increases dramatically near critical transitions or tipping points. To evaluate whether PES effectively captures cellular heterogeneity, we performed dimensionality reduction and clustering analyses based on PES matrix signaling genes, as described in the “Dimension reduction and clustering based on PES” section of the Methods and Materials. For comparison with conventional expression‐based methods, parallel analyses also conducted using the gene expression matrix (GEM) of the top 10% highly variable genes (HVGs). For each dataset mentioned above, as depicted in Figure 4B,C, the PES visualizations of signaling genes demonstrate clearer clustering and separation between different cell types or time points, where cell populations are more clearly delineated compared to the GEM visualizations of HVGs. Moreover, five distinct clustering algorithms, including Hierarchical [55], DBSCAN [56], HDBSCAN [57], K‐Medoids [58], and k‐means [59], are employed to quantitatively compare the clustering performance of PES compared to that of GEM (Figure 4D). The results of the adjusted Rand index (ARI) show that the PES representation of signaling genes outperforms the GEM representation of HVGs, as reflected by higher scores in clustering performance metrics. Analyses of PES‐based dimension reduction and clustering for RD proliferation data are provided in Figure S2. In addition, the UMAP results similarly distinguish cellular states at various time points as effectively as the t‐SNE analysis (Figure S3), which demonstrates that PES‐based representations can robustly capture cell‐state transitions and cellular heterogeneity across different time points, regardless of whether UMAP or t‐SNE is used for visualization. Collectively, these results indicate that PES enhances the resolution and separability of cellular states, providing superior performance in visualizing and characterizing complex and heterogeneous single‐cell data compared with conventional expression‐based representations.

**FIGURE 4:**
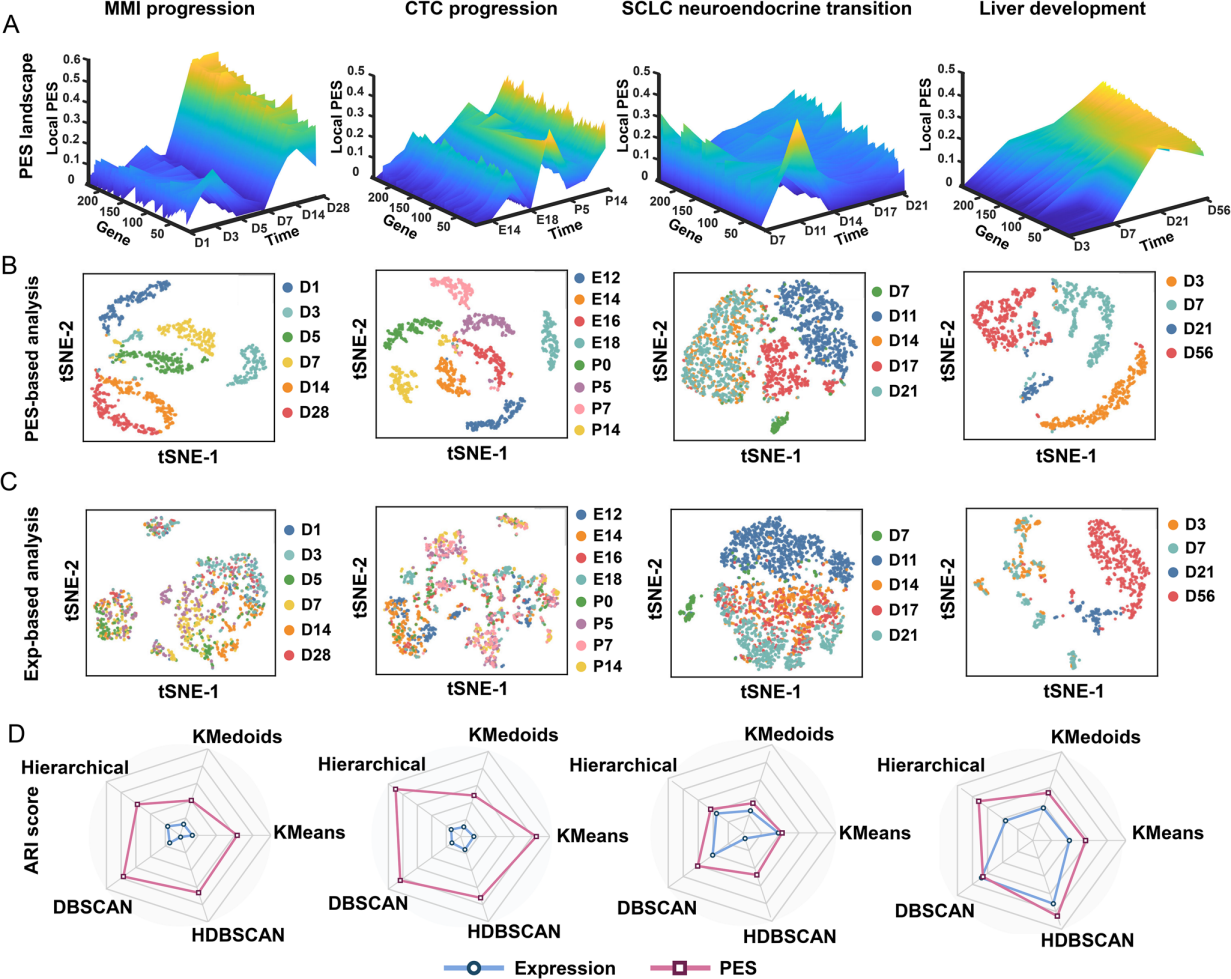
Comparative analysis of the performance of signaling genes' PES and the GEM of HVGs in dimension reduction and clustering. (A) The PES landscape is utilized to illustrate the dynamic variation in the PES of signaling genes from a global perspective across four single‐cell datasets, including MMI progression, CTC progression, SCLC neuroendocrine transition, and liver development. (B) Dimension reduction and clustering analysis are performed for these datasets based on the PES of signaling genes, with different colors representing distinct cell types or time points. (C) Dimension reduction and clustering analysis are conducted out for these datasets based on the GEM of highly variable genes (HVGs). (D) The ARI metric is employed to assess the clustering performance of the PES of signaling genes versus the GEM of HVGs.

### 2.6 Analysis of the Functional Roles for Signaling Genes

To further investigate the involvement of signaling genes in complex biological processes, we carry out a comprehensive analysis of their functional roles. Specifically, the transport process captured by scTIDE from the before‐transition to critical states facilitates the depiction of complex biological progression through distribution patterns derived from manifold‐based graphs. In this study, principal component analysis (PCA) [60] is employed to illustrate the major transformation patterns in the distributions of signaling molecules at various stages of complex biological processes. As shown in Figure 5A–D, for the distribution transport process for four single‐cell datasets (MMI progression, SCLC neuroendocrine transition, liver development, and MP development), demonstrates that the distribution changes are minimal when far from critical transitions but become dramatic near tipping points. Temporal changes in the distribution of the transport process across the entire course of stages or time points are depicted in Figure S4. Moreover, it is seen from Figure 5E–G that certain signaling genes have been identified as upstream transcriptional regulators targeting downstream molecules critical for complex biological progression, thereby enabling the prediction of transcription factors (TFs) that may serve as pivotal regulators of key biological processes. In addition, KEGG enrichment analysis indicates that the identified signaling genes are significantly involved in pathways associated with key biological processes (Figure 5H–J), while GO analysis further corroborates these findings by revealing significant enrichment of biologically relevant functional terms (Figure S5). Several signaling genes obtained from the SCLC neuroendocrine transition and liver development datasets have been reported in the literature to play key roles in the corresponding biological processes (Tables S3 and S4), further supporting the biological significance of our results. Functional analysis of signaling molecules for other single‐cell datasets is presented in Figure S6. Moreover, to predict whether unknown cells reach a tipping point, a neural network model is trained based on the PES profiles of signaling genes derived from observable cells in the non‐critical and critical states (see the “Prediction of the likelihood of critical states in unseen cells” section in the Methods and Materials for details). Additionally, an analogous analytical procedure is carried out using the expression profiles of the top 10% HVGs. It is observed that prediction models constructed using the PES profiles of signaling genes yield high accuracy and show better performance compared with models based on the expression value of HVGs (Figure 5K–L). In terms of precision and recall, models derived from PES profiles of signaling genes also outperform HVG expression‐based methods (Figure S7). scTIDE demonstrates the ability to avert or delay catastrophic outcomes by identifying whether cells are undergoing irreversible transitions, thereby supporting personalized disease diagnosis and treatment.

**FIGURE 5:**
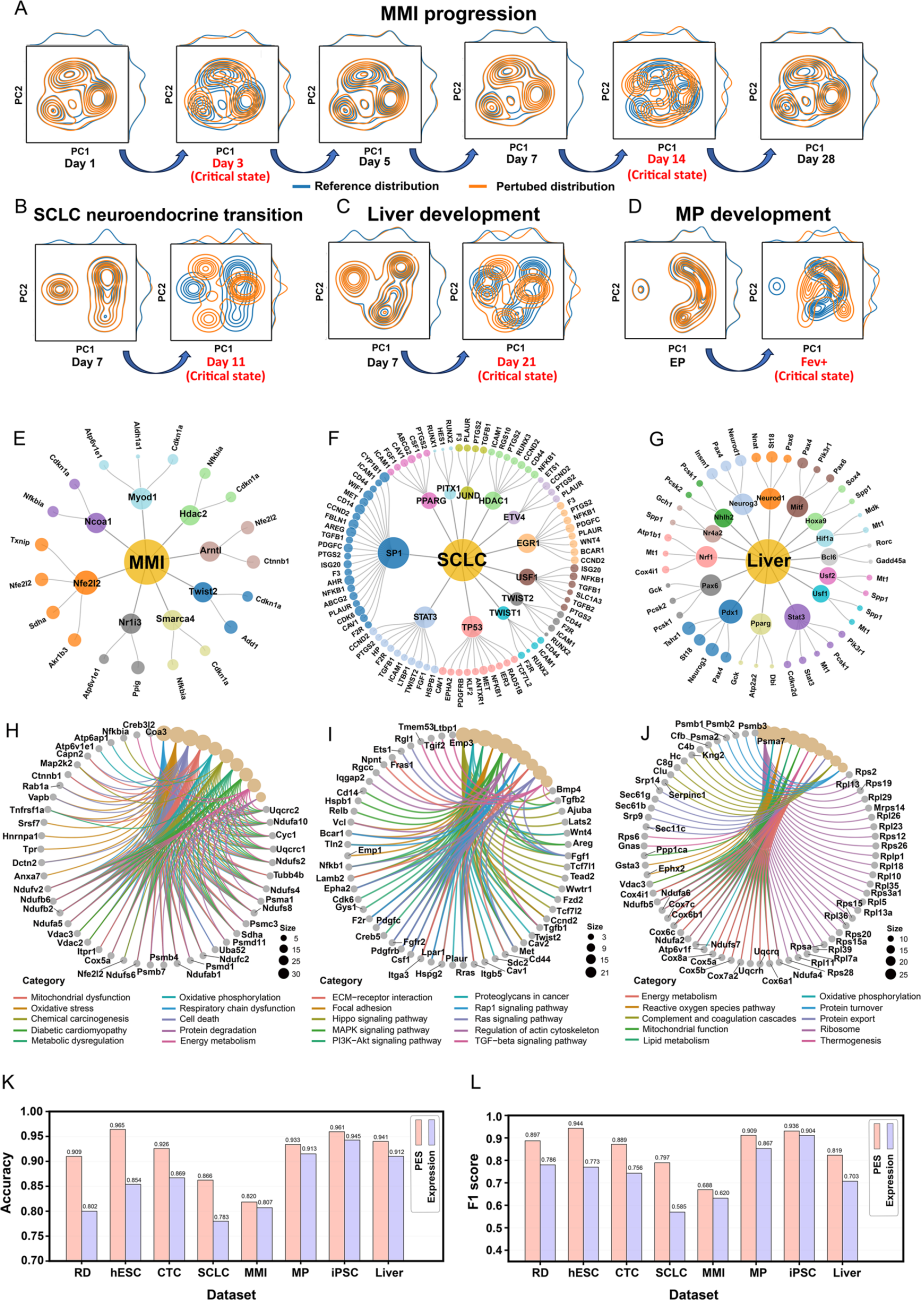
Functional roles of signaling molecules involved in complex biological processes. PCA‐based visualizations of distributional transport from the before‐transition to the critical state across four single‐cell datasets: (A) MMI progression, (B) SCLC neuroendocrine transition, (C) liver development, and (D) MP development. Certain signaling genes function as upstream transcriptional regulators that target downstream molecules involved in key biological progression in three single‐cell datasets: (E) MMI progression, (F) SCLC neuroendocrine transition, and (G) liver development. KEGG pathway enrichment analysis is performed to explore the functions of signaling molecules across three single‐cell datasets: (H) MMI progression, (I) SCLC neuroendocrine transition, and (J) liver development. (K) Prediction accuracies for unseen cells across eight single‐cell datasets and (L) F1 scores of the corresponding predictions, evaluated with consideration of class imbalance.

### 2.7 Potential Signaling Molecule Mechanism for Cell Development and Differentiation

A Monocle‐based trajectory analysis was applied to single cells from various cell types to infer lineage relationships during murine pancreatic (MP) development (Figure 6A). Heatmap analysis of Monocle‐inferred gene expression revealed the temporal sequence of key gene expression events during cell development and differentiation, indicating that sequential changes in the expression of signaling genes may orchestrate MP development (Figure 6B). GO enrichment analysis further reveals that signaling genes are significantly involved in biological processes critical for MP development, such as peptide hormone secretion, insulin secretion, and the regulation of glucose homeostasis (Figure 6C). Moreover, GSVA analysis of the signaling genes was carried out to investigate the dynamic changes in biological functions and pathways during MP development. As shown in Figures 6E and 6D, the GSVA scores for pathways such as Spindle assembly checkpoint signaling, Beta oxidation acyl coa synthesis, and Tight junction actin signaling pathway exhibits an upward trend before the onset of cell fates, while pathways including Aab7 regulated microtubule minus end directed transport, Microtubule rhoa signaling pathway, and Gh jak stat signaling pathway shows a decreasing trend prior to cell fate specification. These findings demonstrate that systemic changes occur before cell fate onset and that transcriptomic profiles of Fev^+^ cells contain essential information for identifying early signs of cell fate decisions.

**FIGURE 6:**
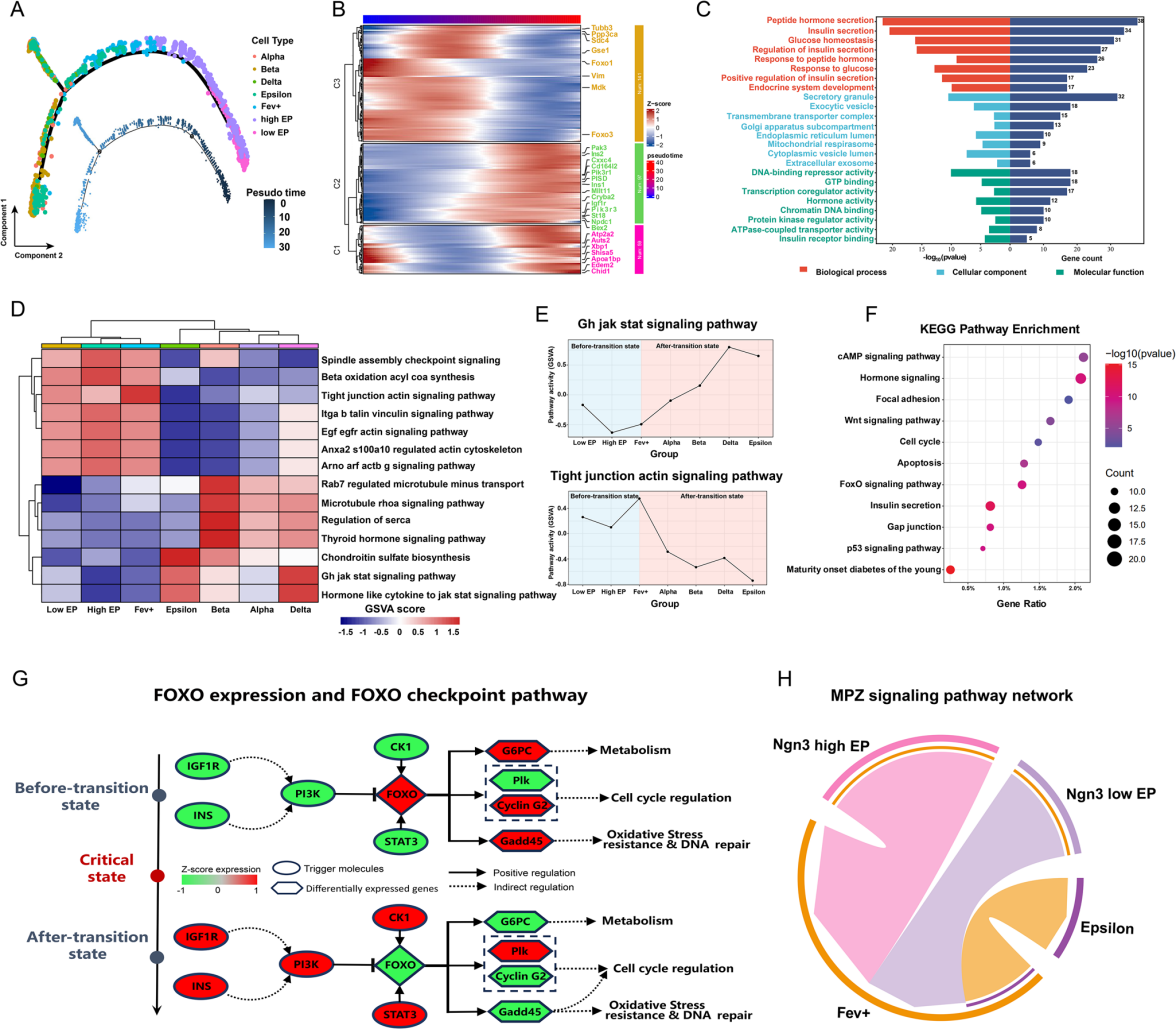
Potential signaling molecule mechanism underlying MP development. (A) Inferred potential trajectories of cell development and differentiation during MP development. (B) The heatmap for dynamic temporal patterns of key gene expression during MP development. (C) Gene Ontology (GO) functional enrichment analysis reveals that the signaling molecules are mainly enriched in MP development–related biological processes. (D) GSVA of the signaling genes was conducted to assess dynamic alterations in biological functions and pathways throughout MP development. (E) GSVA‐score alterations before and after the onset of cell fate transition. (F) KEGG enrichment analysis indicates that the signaling genes are predominantly involved in pathways associated with cell development and differentiation. (G) Functional analysis of upstream signaling molecules and downstream DEGs reveals potential regulatory mechanisms associated with MP development. (H) Cell‐cell communication analysis indicates that the MPZ signaling pathway mediates interactions between Ngn3 Low/High EP and Fev+ cells, as well as between Fev+ and Epsilon cells.

Furthermore, from a KEGG pathway perspective, signaling genes are primarily enriched in pathways related to cell development and differentiation, including the cAMP signaling pathway, hormone signaling, Wnt signaling pathway, and FoxO signaling pathway (Figure 6F). Notably, the FoxO signaling pathway functions as a key regulatory axis governing cell development and differentiation. As illustrated in Figure 6G, the upregulation of upstream signaling molecules, *IGF1R* and *INS*, enhances IGF/insulin signal transduction and activates the PI3K–AKT pathway, which may promote AKT‐mediated nuclear exclusion of FOXO transcription factors, thereby suppressing their transcriptional activity and driving cells toward a terminally differentiated state [61]. Subsequently, Suppression of FOXO activity triggers a comprehensive reorganization of metabolic and cell‐cycle regulatory modules, with downregulation of G6PC signifying a shift from a progenitor‐like metabolic state toward the glucose‐sensing and secretion machinery characteristic of mature endocrine cells [62]. Additionally, most downstream cell‐cycle regulatory molecules (identified as DEGs), including *CyelinG2* and *Gadd45*, are markedly downregulated, facilitating cell‐cycle exit and proliferation arrest to maintain the stable functional state of mature endocrine cells [63]. Thus, such a signaling regulatory mechanism indicates that Fev^+^ subpopulations may undergo extensive metabolic and cell‐cycle remodeling during their transition toward mature endocrine cell identity, enabling adaptation to the terminally differentiated state.

In terms of intercellular communication, the MPZ pathway predicts *Mpzl1*–*Mpzl1* homophilic interactions, primarily occurring between Ngn3 Low/High EP and Fev + cells, as well as between Fev + and Epsilon cells (Figure 6H and Figure S8). *Mpzl1*, an ITIM‐containing membrane receptor‐like molecule, recruits SHP2 upon phosphorylation to form a signaling complex that regulates integrin/Src‐dependent adhesion and migration [64, 65]. These results indicate that interactions between Ngn3 Low/High EP and Fev + cells engage in contact‐dependent adhesion and morphological remodeling to facilitate endocrine lineage differentiation, whereas Fev + and Epsilon interactions reflect the differentiation process following lineage commitment [66].

## 3 Discussion

The capacity to identify key transitional stages during complex biological progression, such as the prodromal phases preceding disease deterioration and the critical decision points in cell lineage commitment, is important for understanding underlying mechanisms and for enabling timely interventions to prevent or postpone adverse outcomes. However, conventional methods are generally inadequate for capturing the temporal dynamics of biological processes and may fail to detect critical signals in real‐world single‐cell datasets typically characterized by substantial noise, sparsity, and cellular heterogeneity. In this paper, we introduce scTIDE, an innovative model that combines manifold‐based graphs with the OT‐CFM framework to uncover critical states and identify signaling molecules in complex biological processes at the individual cell level. Our scTIDE was validated using simulated data and subsequently applied to eight single‐cell datasets across a wide range of biological processes, including disease‐related events such as MMI progression, CTC progression, and SCLC neuroendocrine transitions, as well as developmental processes covering MP development, liver development, hESC differentiation, iPSC differentiation, and RD proliferation. The computational results underscore scTIDE as a powerful tool for quantifying the dynamics of complex biological systems and accurately identifying critical transitions at the individual cell level.

The main strengths of our proposed scTIDE can be summarized as follows. First, by modeling molecular relationships through cell‐perturbed manifold graphs, scTIDE captures distributional changes in local network structure rather than relying solely on coordinate‐wise variation. Second, by integrating these graph‐based representations with optimal‐transport conditional flow matching, scTIDE provides a transport‐based measure of state change that is effective for identifying critical transitions in noisy and high‐dimensional single‐cell data. Third, scTIDE operates at the individual‐cell level relative to a reference population, enabling personalized characterization of critical‐state‐associated instability and the identification of candidate signaling molecules. Finally, the resulting PES representation supports downstream analyses, including classification of unseen cells, dimensionality reduction, and clustering.

Several aspects warrant further exploration in future studies. On the one hand, the current scTIDE framework models system states in a discrete manner and therefore does not sufficiently capture the continuous temporal dynamics underlying complex biological processes. Extending scTIDE to explicitly incorporate continuous‐time modeling or trajectory‐aware representations could enable a more refined characterization of gradual state transitions and critical dynamics. On the other hand, scTIDE could be further augmented by combining with foundation‐model–based frameworks [67], where large‐scale pretraining supports the learning of transferable representations for manifold‐based interaction patterns. Collectively, these future research avenues are expected to advance our understanding of temporal dynamics and underlying mechanisms of complex biological systems while improving the practicality, scalability, and robustness of our proposed framework.

## 4 Methods and Materials

### 4.1 Diffusion‐Informed Manifold‐Based Graphs

To capture the intrinsic geometry of the underlying data manifold, scTIDE constructs diffusion‐informed manifold‐based graphs that characterize intrinsic geometric relationships among genes through the following steps.

#### 4.1.1 Markov Transition Matrix Construction

Manifold learning techniques typically exploit the local Euclidean properties of the manifold, which can be effectively represented by a k‐nearest‐neighbor (kNN) graph. Accordingly, we model local geometric structure using a kNN graph to approximate neighborhood relationships among genes. Specifically, a binary adjacency (similarity) matrix 𝐾 is constructed using a box kernel:

(7) $$K\;\left(g_{i},g_{j}\right)=\left\{\begin{array}{c} 1\;\;\;\;if\;g_{j}\in N_{k}\left(g_{i}\right),\\ 0\;\;\;\;\;\;\;else, \end{array}\right.\;$$

where *N_k_* (g*_i_*) denotes the set of *k* nearest neighbors of *g_i_*, and the PES index consistently identifies the critical state and exhibits similar overall trends across different choices of *k* (ranging from 5 to 15) (Figure S9). This box kernel creates a binary adjacency matrix *K*, where each entry indicates whether two genes form a k‐nearest‐neighbor relationship. Then, to model stochastic transitions on the kNN graph, the adjacency matrix *K* row‐normalized to obtain a Markov transition matrix.

(8) $$M=S^{-1}\;K.$$

Here, *S* is a diagonal degree matrix with entries *S_ii_* = $\sum_{j}K\left(g_{i},g_{j}\right)$. The resulting matrix *M* defines a random walk process on the kNN graph and serves as the diffusion operator in subsequent steps.

#### 4.1.2 Personalized PageRank–Based Diffusion

To capture both local and global manifold structure, we employ a personalized PageRank‐based diffusion process with node‐specific restart probabilities. Let *C*  =  *diag*(*c*_1_,*c*_2_,···, *c_n_*) be a diagonal matrix of damping factors, where *c_i_* ∈ (0,1) controls the balance between diffusion and restart for a node or gene *i*. The diffusion process is defined iteratively as *D* ^(*t*)^ =  *CD* ^(*t* − 1)^ *M* + (*I* − *C*)*M^l^* (as given in Equation 1), where *l* denotes the restart depth (set to *l*  =  2 by default). As *t* → ∞, the process converges to a stationary diffusion state D* (see Section S16), which encodes diffusion‐based similarity profiles for all nodes. Each row of D* corresponds to the steady‐state influence distribution of a query node over the manifold.

#### 4.1.3 Manifold Geodesic Distance

Based on the diffused representation D*, pairwise similarities between nodes are quantified using the Bhattacharyya kernel as follows.

(9) $$F\left(d_{i},d_{j}\right)=<\sqrt{d_{i}},\sqrt{d_{j}}>,$$

where ***d***_***i***_ and ***d***_***j***_​ denote the *i*‐th and *j*‐th rows of D*, respectively. This kernel measures the similarity between diffusion probability distributions and naturally respects the statistical manifold induced by the diffusion process. Moreover, a manifold geodesic distance between nodes *i* and *j* is defined as $MD_{ij}=\sqrt{-2\log F(d_{i},d_{j})}$ (as shown in Equation (2)), which provides a geometry‐aware approximation of the shortest‐path distance along the underlying manifold. The resulting manifold distance matrix can capture both local neighborhood geometry and global topological structure, yielding a robust estimate of intrinsic geodesic distances in high‐dimensional, noisy single‐cell data. Through the above procedure, we construct diffusion‐informed manifold‐based graphs used in downstream distributional modeling and critical transition analysis.

### 4.2 OT‐CFM for Learning Manifold Distribution Dynamics

Our objective is to quantitatively characterize the dynamic differences between the reference distribution (derived from a reference manifold‐based graph) and the perturbed distribution (inferred from a perturbed manifold‐based graph) for a given case cell using OT‐CFM. To this end, we model the evolving topological structure of the system as a time‐varying probability distribution embedded in a manifold‐based graph.

To quantify critical state transitions in the system, we employ optimal transport to compute couplings between the reference distribution and the perturbed distribution. Specifically, let *p*_0_ and *p*_1_ denote the reference and perturbed probability distributions defined on the manifold distance space M, respectively. Optimal transport seeks a joint distribution π(*p*_0_,*p*_1_), referred to as a temporal coupling, that minimizes the expected transport cost between the reference and perturbed distributions subject to marginal constraints. This temporal coupling characterizes how probability mass is transported from one region of the manifold distance space to another during the transition from the reference to the perturbed state. Under this formulation, system evolution is represented as the redistribution of probability mass over the manifold distance space.

To model the continuous evolution of distributions without explicitly simulating transport trajectories, we adopt the flow matching framework. Flow matching is a simulation‐free approach that learns a time‐dependent velocity field by constructing conditional probability paths between the reference and perturbed distributions. Specifically, we define a time‐varying vector field parameterized by a fully connected neural network (multilayer perceptron, MLP) with parameters θ: $v_{\theta }\left(t,x\right):\left(0,1\right)\times M\to T_{M}$, which aims to approximate the true velocity field governing the evolution of the probability distribution. The architecture comprises two hidden layers with 32 neurons each, followed by ReLU activation functions. The network is trained by minimizing the mean squared deviation between the predicted velocity *v*_θ_(*t*, *x_t_*) and the conditional velocity *u_t_* (*x*|*z*) along transport paths, as defined by the OT‐CFM loss $L_{OT-CFM}$ in Equation (4), with a fixed noise scale of ε = 0.1, while optimization is performed using the Adam optimizer with a learning rate of 1 × 10^−3^. Here, *z*  =  (*x*_0_,*x*_1_) denotes a source–target sample pair drawn from the OT coupling π(*p*_0_,*p*_1_). A detailed description of the symbol, model architecture, and training setting used in the OT‐CFM module is provided in Table S5. Although the true velocity field *u_t_* (*x*) is generally unknown, minimizing the OT‐CFM loss is gradient‐equivalent to minimizing the expected deviation between *v*_θ_(*t*, *x_t_*) and *u_t_* (*x*). Consequently, the optimal network $v_{\theta^{*}}\left(t,x\right)$ approximates the posterior mean velocity field *u_t_* (*x*). This learned velocity field provides a continuous description of manifold distribution dynamics while avoiding explicit trajectory simulation.

### 4.3 Deciphering Critical Transitions via scTIDE

Based on a population of reference cells derived from a relatively stable or healthy state, scTIDE can be utilized to detect critical transitions or tipping points at the individual‐cell level. The detailed procedure is given in the following steps.

**(Step 1) Construction of reference and perturbed manifold‐based graphs**. We construct the reference manifold‐based graph *MG*_Re_ for the background/reference cells and perturbed manifold‐based graph *MG*_Per_ for the mixed cells (comprising both an individual case cell and the reference cell population). Specifically, for the reference cells, the Markov transition matrix *M*_Re_ is computed according to Equations (7) and (8). Moreover, based on *M*_Re_, the reference manifold‐based graph *MG*_Re_ is constructed following Equations (1) and (2). Similarly, the perturbed manifold‐based graph *MG*_Per_ is constructed using the same procedure applied to the mixed cell set.

**(Step 2) Extraction of local manifold‐based graphs**. From the global manifold‐based graph, we extract gene‐centered local manifold‐based graphs. Specifically, for a given gene *g^k^* in the reference manifold‐based graph *MG*_Re_, its *g^k^*‐local manifold‐based graph LMGRek can be extracted, which is centered around the *g^k^* and includes its first‐order neighbors $\;\{g_{1}^{k},\;\;g_{2}^{k},\text{…},\;g_{U}^{k}\}$. The geodesic distances on manifold between the central gene *g^k^* and its neighbors, denoted as {MDRe(gk,g1k),MDRe(gk,g2k),…,MDRe(gk,gUk)}, are determined by the manifold distance index *MD_ij_*, as defined as the Equation (2). Consequently, if there are *N* genes in the reference manifold‐based graph *MG*_Re_, *N* distinct local manifold‐based graphs LMGRek(k=1,2,…,N) can be generated. Similarly, for the perturbed manifold‐based graph *MG*_Per_, the corresponding *g^k^*‐local manifold‐based graphs $LMG_{\mathrm{Per}}^{k}\;\left(k=1,2,\text{…},N\right)$ can be extracted, with manifold geodesic distances expressed as $\left\{MD_{\mathrm{Per}}\left(g^{k},g_{1}^{k}\right),MD_{\mathrm{Per}}\left(g^{k},g_{2}^{k}\right),\text{…},MD_{\mathrm{Per}}\left(g^{k},g_{U}^{k}\right)\right\}$.

**(Step 3) Estimation of reference and perturbed distributions**. For each local manifold‐based graph, we estimate a reference distribution and a perturbed distribution. Specifically, for the reference *g^k^*‐local manifold‐based graph LMGRek, the reference distribution PRek is constructed based on the manifold geodesic distances between the central gene *g^k^* and its neighbors $\;g_{i}^{k}$ (*i*  =  1, 2, ···, *U*), as described below.

(10) PRek=p1kp2k⋮pUk,pik=MDRegk,gik∑j=1UMDRegk,gjk

Similarly, for the perturbed *g^k^*‐local manifold‐based graph $LMG_{\mathrm{Per}}^{k}$, the perturbed distribution $\;Q_{\mathrm{Per}}^{k}$ is defined as:

(11) $$Q_{\mathrm{Per}}^{k}=\left[\begin{array}{c} \begin{array}{c} q_{1}^{k}\\ q_{2}^{k}\\ \vdots \end{array}\\ q_{U}^{k} \end{array}\right]\;,\;q_{i}^{k}=\frac{MD_{\mathrm{Per}}\left(g^{k},g_{i}^{k}\right)}{\sum_{j=1}^{U}MD_{\mathrm{Per}}\left(g^{k},g_{j}^{k}\right)}\;.$$

For both distributions PRek and $\;Q_{\mathrm{Per}}^{k}$, it is evident that $\;\;\sum_{i=1}^{U}p_{i}^{k}=1$ and $\sum_{i=1}^{U}q_{i}^{k}=1.$

**(Step 4) Computation of the cell‐specific path energy score (PES)**. For each individual cell *C*_*t* _​ at time point *t*, we compute its path energy score (PES), denoted as *E*_*t* _. First, for each *g^k^*‐local manifold‐based graph, the local PES *E^k^* is calculated from the corresponding reference and perturbed distributions PRek and $\;Q_{\mathrm{Per}}^{k}$ using Eqs. (3)‐(6). Then, the cell‐specific PES *E*_*t* _ is obtained by averaging the local PES values over a subset of local graphs exhibiting the highest energy scores:

(12) $$E_{t}=\frac{1}{L}\;\sum_{k=1}^{L}E^{k},\;$$

where *L* is a configurable parameter representing the top 10% of *g^k^*‐local manifold‐based graphs ranked by *E^k^*. Our analysis indicates that scTIDE consistently deciphers the same critical transitions and exhibits similar PES patterns across parameter *L* ranging from 5% to 15% (Figure S10). As the system approaches a critical transition, the probability distribution at the critical state deviates substantially from that of the before‐transition state, resulting in a pronounced increase in the PES.

**(Step 5) Identification of critical transitions**. To determine whether scTIDE captures statistically significant transitions, we apply a two‐sample Student's *t*‐test [68] to compare the PES values between consecutive states. Specifically, the two‐sample t‐statistic *Z* is used to assess whether the mean of an *n*_1_‐dimensional vector $\hat{X_{1}}=\left(x_{1}^{1},x_{1}^{2},\text{…},x_{1}^{n_{1}}\right)$ deviates significantly from the mean of an *n*_2_‐dimensional vector $\hat{X_{2}}=\left(x_{2}^{1},x_{2}^{2},\text{…},x_{2}^{n_{2}}\right)$, that is,

(13) $$Z=\frac{\mathrm{mean}\left(\hat{X_{1}}\right)-\mathrm{mean}\left(\hat{X_{2}}\right)}{\sqrt{\frac{\mathrm{Var}\left(\hat{X_{1}}\right)}{n_{1}}+\frac{\mathrm{Var}\left(\hat{X_{2}}\right)}{n_{2}}}}\;,$$

where $\mathrm{mean}(\hat{X_{1}})$ and $\mathrm{mean}(\hat{X_{2}})$ represent the means of the vectors $\hat{X_{1}}$ and $\hat{X_{2}}$, respectively, while $\mathrm{Var}(\hat{X_{1}})$​ and $\mathrm{Var}(\hat{X_{2}})$ denote their variances. The *P*‐value associated with the *Z* statistic is used to assess the significance of the difference between the means of the vectors $\hat{X_{1}}$ and $\hat{X_{2}}$. A *P*‐value less than or equal to 0.05 indicates a statistically significant difference between $\mathrm{mean}(\hat{X_{1}})$ and $\mathrm{mean}(\hat{X_{2}})$. For each time point *t*, the PES values of all cells are collected into a vector $\;\hat{E_{t}}$. In this study, a time point *t* ≥ 2 is classified as in a critical state if the following criteria are satisfied: (i) the mean of the vector $\hat{E_{t}}$ is significantly greater than that of ${\hat{E}}_{t-1}$(*P*‐value ≤ 0.05), indicating a statistically significant increase relative to the preceding state; and (ii) the mean of the vector ${\hat{E}}_{t+1}$ does not exhibit a significant increase compared to ${\hat{E}}_{t}$ (*P*‐value > 0.05), suggesting the absence of a statistically significant increase relative to the subsequent state. In addition, we apply Cohen's d (CSD) to evaluate whether the difference between the critical state and the before‐transition state is of practical significance, providing a measure of effect size beyond statistical significance (see Section S20).

### 4.4 Dimension Reduction and Clustering Based on PES

scTIDE transforms sparse gene expression data into a non‐sparse path energy score (PES) matrix using Equations (3)–(6). The resulting PES matrix preserves the same structure as the original gene expression matrix (GEM), with rows corresponding to genes and columns representing cells. Consequently, the PES matrix can be directly analyzed using standard scRNA‐seq pipelines for dimensionality reduction and cell clustering by replacing the original GEM. To evaluate the ability of PES‐derived signaling genes to capture cellular heterogeneity and dynamic transitions, the top 10% of genes exhibiting the highest local PES values at the critical state were selected as candidate signaling genes for subsequent PES‐based clustering analysis. The t‐distributed stochastic neighbor embedding (t‐SNE) [69] was applied to perform nonlinear dimensionality reduction and visualization for the PES matrix of signaling genes. Specifically, the reduced representations for t‐SNE visualization and analysis were generated based on the top 10% signaling genes over the PES value.

To quantitatively assess clustering performance, we applied five clustering algorithms, including KMedoids, KMeans, HDBSCAN, DBSCAN, and hierarchical clustering, to the PES‐based representations. Clustering accuracy was evaluated using the Adjusted Rand Index (ARI), which measures the agreement between predicted cluster assignments and ground‐truth partitions. By correcting for chance agreement through pairwise assignment analysis, the ARI provides a robust evaluation metric, where higher values indicate more accurate clustering and a more faithful recovery of the underlying data structure. The ARI is defined as:

(14) $$\mathrm{ARI}\;=\frac{\sum_{i,j}\left(\begin{array}{c} n_{ij}\\ 2 \end{array}\right)-\left[\sum_{i}\left(\begin{array}{c} a_{i}\\ 2 \end{array}\right)\sum_{j}\left(\begin{array}{c} b_{j}\\ 2 \end{array}\right)\right]/\left(\begin{array}{c} n\\ 2 \end{array}\right)}{\frac{1}{2}\left[\sum_{i}\left(\begin{array}{c} a_{i}\\ 2 \end{array}\right)+\sum_{j}\left(\begin{array}{c} b_{j}\\ 2 \end{array}\right)\right]-\left[\sum_{i}\left(\begin{array}{c} a_{i}\\ 2 \end{array}\right)\sum_{j}\left(\begin{array}{c} b_{j}\\ 2 \end{array}\right)\right]/\left(\begin{array}{c} n\\ 2 \end{array}\right)}\;$$

where *n* is the number of cells, *n_ij_* represents the number of cells simultaneously assigned to the cluster *i* in the ground‐truth clustering and cluster *j* in the predicted clustering, and *a*_*i* _and *b_j_* denote the marginal totals of cells in the cluster *i* (ground‐truth clustering) and cluster *j* (predicted clustering), respectively.

### 4.5 Predicting the Likelihood of Critical States in Unseen Cells

Cells are categorized into critical and non‐critical states based on the identified transition points. To evaluate the predictive capability of scTIDE, we train a deep learning classifier to predict whether unseen cells belong to the critical state using the PES profiles of signaling genes. The overall workflow is outlined below.

#### 4.5.1 Batch‐Effect Correction

To eliminate systematic technical variability across experimental batches, batch‐effect correction is performed using the ComBat algorithm implemented in pyComBat [70]. This procedure removes non‐biological variance while preserving intrinsic signaling patterns, thereby generating harmonized PES matrices suitable for downstream predictive modeling.

#### 4.5.2 Adaptive Class Weighting for Imbalanced Training Data

To address class imbalance and maintain sensitivity to minority critical‐state cells, an adaptive class‐weighting strategy is incorporated during training. Specifically, given the predominance of non‐critical cells over critical cells, the critical class is assigned an increased weight (*w*_1_ =  2), whereas the non‐critical class retains a baseline weight (*w*_0_ =  1). This strategy mitigates majority‐class dominance and stabilizes model convergence under skewed class distributions.

#### 4.5.3 Neural Network Architecture and Loss Optimization

A deep neural network classifier is constructed to estimate the probability that an unseen cell belongs to the critical state. The model consists of a feature extractor followed by a classification module. The feature extractor is composed of stacked fully connected layers, each including a linear transformation, Batch Normalization, LeakyReLU activation, and Dropout regularization to learn robust latent representations. The classification module contains four fully connected layers with progressively decreasing hidden dimensions, each followed by Batch Normalization and Leaky ReLU activation. Dropout is applied to the final two layers to mitigate overfitting and enhance generalization. The model can be formulated as:

(15) $$Z=f_{\mathrm{extractor}}\;\left(x;\theta_{e}\right),$$

(16) $$\hat{y}=f_{\mathrm{classifier}}\;\left(Z;\theta_{c}\right),$$

where *x* denotes the input feature vector consisting of the PES values of signaling genes for each cell, *Z* represents the learned latent embedding generated by the feature extractor parameterized by θ_*e*_, and $\hat{y}$ is the predicted probability produced by the classifier parameterized by θ_*c*_. To further compensate for class imbalance, a balanced cross‐entropy loss was adopted:

(17) $$L_{BalancdCE}\;\left(y,\hat{y}\right)=-\sum_{j=0}^{1}w_{j}y_{j}\log\left({\hat{y}}_{j}\right).$$

Here, *w_j_* denotes the class‐specific weighting factor, *y_j_* is the ground‐truth class label, ${\hat{y}}_{j}$ represents the predicted probability for the class *j*. This weighted loss function mitigates the influence of class imbalance by increasing the contribution of minority‐class samples to the overall objective, thereby promoting stable optimization and enhancing the classifier's sensitivity to critical‐state cells while maintaining discriminative performance.

### 4.6 Data Processing and Functional Assessment

To demonstrate the effectiveness and reliability of scTIDE, we applied it to a numerical simulation and eight diverse real‐world single‐cell datasets, covering disease progression‐related contexts such as mouse myocardial infarction (EMBL: E‐MTAB‐7895), cerebellar tumor cells progression (GEO: GSE118068), and neuroendocrine transitions in small cell lung cancer (GEO: GSE149179), as well as developmental processes including murine pancreatic development (GEO: GSE132188), liver development (GEO: GSE171993), human embryonic stem cell differentiation (GEO: GSE75748), induced pluripotent stem cell differentiation (PMCID: PMC5338498), and radial progenitor proliferation (GEO:GSE107122). The selection of these datasets was based on two criteria. First, all of them contain temporal information or dynamic cellular trajectories that describe complete biological processes. Second, they provide the essential information needed to indicate key landmarks of these processes, such as key transitions from experimental observations. Detailed data descriptions and cell sampling information are provided in Section S20. For these single‐cell datasets, probes that did not correspond to the relevant NCBI Entrez gene symbols were removed. For genes matched by multiple probes, expression values were averaged across the mapped probes. In our study, the top 2000–3000 highly variable genes were selected for analysis, which is a widely used strategy in scRNA‐seq workflows to identify the gene set with the greatest informational value. We performed sensitivity analyses with different numbers of selected HVGs (including 1500, 2000, 2500, and 3000 genes), and found that scTIDE is robust to moderate changes in HVGs number selection (Figure S11), demonstrating the stability of the proposed framework. Cells from the earliest time point can be treated as the reference group, representing the relatively healthy or undifferentiated state. Specifically, we select cells from the first time point as the reference group, which corresponds to early‐stage conditions and is generally presumed to reflect a more stable, healthy state before any observable transitions or perturbations occur.

Pathway analysis was performed using the Kyoto Encyclopedia of Genes and Genomes (https://www.kegg.jp), while enrichment analysis was carried out based on Metascape and the ClusterProfiler package [71]. Functional annotations were derived from resources provided by the Gene Ontology Consortium (http://geneontology.org) and Ingenuity Pathway Analysis software. Networks were visualized using Cytoscape (www.cytoscape.org).

## Author Contributions

R.L. and PC conceived the research. J.Y.Z., B.W.N., Y.B.Y., S.Y.N., and XRG performed the real data analysis. All authors wrote the paper. All authors read and approved the final manuscript.

## Conflicts of Interest

The authors declare no conflicts of interest.

## Supporting information




**Supporting File**: advs76606‐sup‐0001‐SuppMat.docx.

## Data Availability

To comprehensively evaluate the proposed method, eight real‐world single‐cell datasets were collected from public repositories, including MMI progression (EMBL: E‐MTAB‐7895) from the BioStudies database, as well as CTC progression (GEO: GSE118068), SCLC neuroendocrine transition (GEO: GSE149179), MP development (GEO: GSE132188), liver development (GEO: GSE171993), hESC differentiation (GEO: GSE75748), iPSC differentiation (PMCID: PMC5338498), and RD proliferation (GEO: GSE107122) from the GEO database (http://www.ncbi.nlm.nih.gov/geo/). The source code of the algorithm and related data are available at https://github.com/Terry‐NIU/scTIDE.
