# Supplementary material for: scTIDE: Deciphering Critical Transitions Through Cell‐Perturbed Manifold Graphs and Optimal Transport Conditional Flow Matching
Source: Adv Sci (Weinh). 2026 Jul 20:e76606. Online ahead of print. doi: 10.1002/advs.76606 (PMC13384040; doi:10.1002/advs.76606)
Supplement: Supplementary file 1 — Supporting File: advs76606‐sup‐0001‐SuppMat.docx. [file ADVS-9999-e76606-s001.docx]

**Supplementary Information: scTIDE: deciphering critical transitions through cell-perturbed manifold-based graph and optimal transport conditional flow matching**

**Contents**

**A. Main properties of dynamic network biomarker (DNB) theory** S2

**B.** **Overview of the dynamical system used for numerical simulations** S3

**C.** **Time points of identified critical states and reported biological observations** S6

**D.** **Performance comparison of scTIDE and the KL-divergence-based variant** S7

**E.** **Comparison of critical point detection methods and implementation details** S8

**F. Evaluation of PES-based dimension reduction and clustering performance** S9

**G.** **Distributional transport map of signaling molecules** S10

**H.** **Functional analysis of signaling molecule** S11

**I.** **Robustness analysis of scTIDE with respect to the kNN parameter** $\boldsymbol{k}$ S16

**J.** **Derivation of the diffused representation** S16

**K. Description of model architecture and training settings in the OT-CFM module**  S18

**L.** **Performance of scTIDE under varying parameter** $\boldsymbol{L}$ S19

**M.** **Effect size quantification of critical states using Cohen’s d (CSD)** S20

**N.** **Description of the eight real single-cell datasets** S20

**O.** **Stability analysis of scTIDE with respect to the number of HVGs** S22

1. **Main properties of dynamic network biomarker (DNB) theory**

Our recently proposed dynamical network biomarker (DNB) theory posits that, as a complex system approaches a tipping point, a specific subset of molecules, defined as the DNB, displays enhanced correlations and synchronized fluctuations in their behavior [1]. Specifically, the discrete-time dynamical system describing the evolution of the network can be written as follows:

$Z\left( t+1 \right)=f\left( Z\left( t \right);P \right),$ (S1)

where $Z(t)=(z_{1}(t),z_{2}(t),\cdots\text{ },z_{n}(t))$ denotes an $n$-dimensional state vector at time step $t$, representing the system features, and $P=(p_{1},p_{2},\cdots\text{ },p_{s})$ is the parameter vector describing the slowly varying driving factors. In general, the mapping $f:\mathbb{R}^{n}\times\mathbb{R}^{s}\to\mathbb{R}^{n}$ is assumed to be nonlinear.

For the dynamical system in Eq. (S1) with parameter $P$, a bifurcation or critical transition arises when the following conditions are satisfied:

1. $\bar{Z}$ represents a fixed point of the system, defined by the condition $\bar{Z}=f(\bar{Z};P)$.

2. There exists a value $P_{c}$ such that one or a pair of eigenvalues of the Jacobian matrix $\frac{\partial f(Z;P)}{\partial Z}|_{Z=\bar{Z}}$ has modulus equal to 1.

3. When $P\neq P_{c}$, the eigenvalues of the linearized function of $f$ generally do not have a modulus equal to 1.

Together with the corresponding transversal conditions, these requirements indicate that the system undergoes a phase transition at $\bar{Z}$, corresponding to a codimension-one bifurcation when $P$ reaches $P_{c}$. For Eq. (S1), before $P$ approaches $P_{c}$, the system is assumed to remain near a stable fixed point $\bar{Z}$, where all eigenvalues have modulus between 0 and 1. The parameter value $P_{c}$ associated with the qualitative change of the system state is referred to as the bifurcation value or critical transition point.

The general dynamical properties of Eq. (S1) can be obtained by analyzing its linearized form together with the influence of stochastic perturbations near $\bar{Z}$. By introducing a new variable $Y(t)=(y_{1}(t),\ldots,y_{n}(t))$and a transformation matrix $S$, such that $Y(t)=S^{-1}(Z(t)-\bar{Z})$, the system can be rewritten as

$Y(t+1)=\Lambda(P)Y(t)+\zeta(t),$ (S2)

where $\Lambda(P)$denotes the diagonalized form of ${\frac{\partial f(Z;P)}{\partial Z}\mid}_{Z=\bar{Z}},$ and $\zeta(t)=(\zeta_{1}(t),\ldots,\zeta_{n}(t))$represents small Gaussian noise terms with zero mean. Let $\sigma_{i}$ denote the small standard deviation of $\zeta_{i}$. Without loss of generality, we consider the diagonalized matrix $\Lambda=(\lambda_{1},\ldots,\lambda_{n})$, where each $\lambda_{i}$ lies in the interval $(0,1)$. In practice, three typical cases may arise in the diagonalization process; a detailed derivation in the ideal case is provided in the reference [2].

Among all eigenvalues of $\Lambda$, the one with the largest modulus, denoted by $\lambda_{1}$, gradually approaches 1 as the parameter $P\to P_{c}$. The eigenvalue $\lambda_{1}$ characterizes the local dynamical behavior of the system near the fixed point and is referred to as the dominant eigenvalue. A pre-transition state corresponds to $\mid\lambda_{1}\mid<1$, whereas the critical regime is reached when $\lambda_{1}$ approaches 1. Without loss of generality, we associate the first component $y_{1}$ in $Y$ with $\lambda_{1}$, such that $\left( y_{1},0,\cdots\text{ },0 \right)$ represents the eigenvector corresponding to $\lambda_{1}$.

Near the fixed point, it has been shown that a dominant group of variables, referred to as a dynamic network biomarker (DNB), emerges and satisfies the following conditions as the system approaches a critical point prior to the transition [1]. The critical properties of the stochastically perturbed linear system described by Eq. (S2) can be summarized as follows. Here, $z_{i}$ in Eq. (S1) denotes the value of feature $i$, such as the expression level of a gene or protein. When $P$ approaches the bifurcation point, the following properties are observed:

- The variability of each molecule within the DNB increases sharply;
- The correlations among molecules within the DNB are significantly enhanced;
- The correlations between DNB molecules and those outside the group are weakened.

The DNB properties indicate that, near the critical point, a group of highly fluctuating and strongly correlated biomolecules with pronounced cooperative interactions serves as an early-warning signal of an imminent transition. Actually, qualitative state transitions in complex biological systems can be detected by analyzing the evolution of such dominant variables and their molecular associations at the network level.

**B. Overview of the dynamical system used for numerical simulations**

To demonstrate the effectiveness of scTIDE, we conducted a numerical simulation using an 18-node regulatory network, as illustrated in Figure 2A of the main text. This modulated network is modeled by a framework of stochastic differential equations based on Michaelis-Menten or Hill dynamics, commonly employed to analyze gene regulation in biological processes, including transcription, translation, and complex nonlinear interactions [3, 4]. Specifically, the dynamics of this 18-node network are described by the following set of 18 differential equations.

(S3)

where $p$ is a scalar parameter, and $\zeta_{i}(t)$ (*i* = 1, 2, $\cdots$, 18) represents Gaussian noise with a mean of zero and covariance given by $k_{ij}=\mathrm{Cov}(\zeta_{i},\zeta_{j})$. The mRNA-i concentrations are described by the molecule $z_{i}(t)$ ($i$ = 1, 2,$\cdots$,18). In Eq.(S3), the degradation rates of mRNAs are represented as$R\text{=}\left(\frac{(4+4|p|)}{15}\text{,}\frac{(8+2|p|)}{15}\text{,1,}\frac{6}{5}\text{,}\frac{7}{5}\text{,}\frac{8}{5}\text{,}\frac{9}{5}\text{,2,}\frac{11}{5}\text{,}\frac{12}{5}\text{,}\frac{13}{10}\text{,}\frac{14}{10}\text{,5,}\frac{16}{10}\text{,}\frac{7}{2}\text{,}\frac{7}{2}\text{,}\frac{19}{5}\text{,4}\right).$ The stable equilibrium point of the dynamic system is denoted as $\bar{Z}=(\bar{z}_{1},\bar{z}_{2},\ldots,\bar{z}_{18})=(0,0,\ldots,0)$. By applying the Euler method, Eq. (S3) can be discretized into a corresponding set of equations using a small time step$\Delta t$.

(S4)

Where $Z(k)$ refers to the vector $Z(t)$ at the time instant $k\Delta t$. The Jacobian matrix for Eq. (S4) is indicated as $J=\left.\frac{\partial f(Z(k);p)}{\partial Z}\right|_{Z=\bar{Z}}$, $J=e^{\Delta t\bullet A}$. Setting $\Delta t=1$ allows for the derivation of eight distinct eigenvalues from Eq. (S4). The dominant eigenvalue satisfies the condition $0.68^{|p|}$→ 1 when $p$→ 0. The special parameter value $p=0$ is corresponds to the bifurcation parametric value. Specifically, for the simulation model governed by the differential equations given in Eq. (S3), a bifurcation occurs at $p=0$. When $p<0$, the modulus of all eigenvalues of the Jacobian matrix of Eq. (S4), evaluated at the equilibrium point, is less than 1, which means that originally the equilibrium point $\left( \overline{z}_{1},\overline{z}_{2},\cdots,\overline{z}_{18} \right)=(0, 0, \cdots, 0)$ is stable. When the parameter $p$ tends to 0, the modulus of the largest eigenvalue approaches 1, which means that the original stable equilibrium $\left( \overline{z}_{1},\overline{z}_{2},\cdots,\overline{z}_{18} \right)$ becomes gradually unstable and further bifurcates into a qualitatively different periodic solution (actually, a Hopf bifurcation occurs). Such a bifurcation behavior represents a phase transition in the system.

**C. Time points of identified critical states and reported biological observations**

We evaluated the performance of scTIDE on eight representative single-cell datasets spanning a diverse range of biological processes. These datasets include disease progression contexts, such as mouse myocardial infarction (MMI) progression [5], cerebellar tumor cell (CTC) progression in childhood [6], and neuroendocrine transitions in small cell lung cancer (SCLC) [7], as well as developmental processes, including murine pancreatic (MP) development [8], liver development [9], human embryonic stem cell (hESC) differentiation [10], induced pluripotent stem cell (iPSC) differentiation [11], and radial progenitor (RD) proliferation [12]. Across all datasets, scTIDE successfully identified critical-transition states that are well consistent with reported biological observations. The time points associated with key reported biological observations and the critical states identified by scTIDE are summarized in Table S1.

**Table S1. Time points of critical states identified by scTIDE and reported biological observations**

| **Dataset** | **Dataset ID** | **Detected transition phase** | **Known biological observations** | **Early-warning lead time** |
| --- | --- | --- | --- | --- |
| **SCLC progression** | GSE149179 | Day 11  (*P* =2.07E-14) | Day 14 | + 1 stages |
| **MMI**  **progression** | E-MTAB-7895 | Day 3  (*P* =2.33E-3)  Day 14  (*P* =1.31E-18) | Day 7  Day 28 | + 2 stages  + 1 stages |
| **RD**  **proliferation** | GSE107122 | E15.5  (*P* =2.50E-26) | E17.5 | + 1 stages |
| **Liver development** | GSE171993 | Day 21  (*P* =1.88E-13) | Day 56 | + 1 stages |
| **CTC progression** | GSE118068 | E16  (*P* =9.88E-8)  P0  (*P* =1.33E-21) | E18  P14 | +1 stages  +3 stages |
| **iPSC differentiation** | PMC5338498 | Day 2  (*P* =2.32E-25) | Day 3 | +2 stages |
| **MP development** | GSE132188 | Fev+  (*P* =2.97E-207) | Differentiation into Alpha, Beta, Delta, Epsilon | +1 stages |
| **hESC differentiation** | GSE75748 | 36 h  (*P* =4.99E-5) | 72 h | +1 stages |

**D. Performance comparison of scTIDE and the KL-divergence-based variant**

An ablation study was conducted by replacing the OT-CFM module with a simpler distributional discrepancy measure based on Kullback–Leibler (KL) divergence. Specifically, for each local graph, we computed the KL divergence between the reference distribution and the perturbed distribution and used it to construct a KL-divergence-based variant. The resulting warning signals were then compared with those obtained by the proposed scTIDE framework. As shown in Figure S1, although the KL-divergence-based variant is able to capture some dynamic changes, it produces weaker and less distinct signals around the critical-transition state. In contrast, the proposed scTIDE framework consistently generates more pronounced PES peaks and more accurately identifies the critical-transition points. These results indicate that directly measuring distributional discrepancies is insufficient to fully characterize the underlying transition dynamics, whereas the OT-CFM module provides additional information by explicitly modeling the transport process between distributions. Thus, the observed performance improvement is not merely due to measuring distributional divergence but also stems from the transport dynamics learned by OT-CFM.


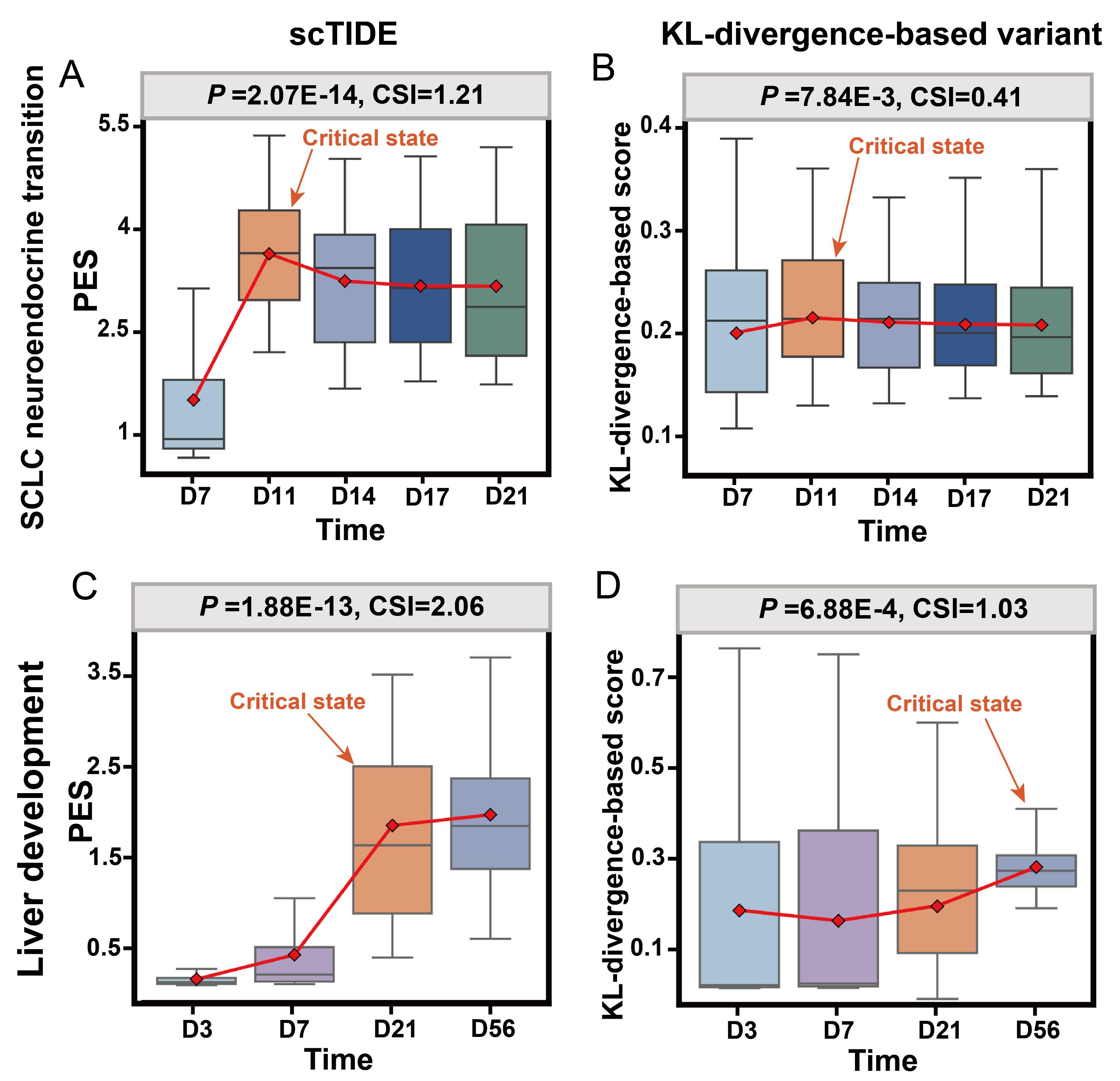


Figure S1. Comparison of warning signals generated by the proposed scTIDE and a KL-divergence-based variant on the (A) SCLC neuroendocrine transition data and (B) liver development data. Compared with the KL-divergence-based variant, our proposed OT-CFM-based scTIDE yields stronger warning signals and achieves more accurate detection of critical-transition states.

**E.** **Comparison of** **critical point detection methods and** **implementation details**

In this study, we compared the proposed scTIDE with several existing critical point detection methods, including directed network flow entropy (DNFE) [13], single-sample landscape entropy (SLE) [14], Gaussian graphical optimal transport (GGOT) [15], module-based dynamic network biomarker (M-DNB) [16], and sample-perturbed Gaussian graphical model (sPGGM) [17]. Specifically, for each method, the algorithms were implemented following the procedures described in the original publications. Unless otherwise specified, default parameter settings were adopted to maintain consistency with the methodological frameworks. All methods were applied to the same preprocessed datasets to ensure comparability, with normalization and feature selection performed consistently across methods. Additional implementation details, including data preprocessing protocols, parameter configurations, and computational runtime environment (e.g., software versions and hardware specifications), are summarized in the following Table S2.

**Table S2. Implementation details of different critical point detection methods**

| **Method** | **Implementation** | **Data**  **preprocessing** | **Runtime environment** | **Github link** |
| --- | --- | --- | --- | --- |
| scTIDE | Python  code (ours) | Log (x + 1) transformation | Python $\geq$ 3.10,  NVIDIA CUDA with compatible PyTorch version  64GB RAM | https://github.com/Terry-NIU/scTIDE |
| DNFE | Author-provided  MATLAB code | Log (x + 1) transformation | MATLAB $\geq$ 2017a,  64GB RAM | https://github.com/Peng-xq/DNFE |
| SLE | Author-provided  MATLAB code | Log (x + 1) transformation | MATLAB $\geq$ 2010b,  64GB RAM | https://github.com/rabbitpei/SLE |
| GGOT | Author-provided  Python code | Log (x + 1) transformation | Python $\geq$ 3.11,  64GB RAM | https://github.com/huawenbo/GGOT |
| M-DNB | Author-provided  MATLAB code | Log (x + 1) transformation | MATLAB $\geq$ 2017a,  64GB RAM | https://github.com/LinLi-0909/M-DNB-model |
| sPGGM | Author-provided  MATLAB code | Log (x + 1) transformation | MATLAB $\geq$ 2021b,  64GB RAM | https://github.com/Junxian-Li-0/sPGGM_project |

**F.** **Evaluation of PES-based dimension reduction and clustering performance**

To validate the effectiveness of the PES-based representation derived from scTIDE-identified signaling molecules, we evaluated its performance in standard single-cell analysis tasks. Instead of directly using raw gene expression levels, the PES framework replaces the original gene expression matrix (GEM) with the PES matrix, thereby enabling seamless integration into conventional single-cell workflows, including dimensionality reduction and clustering.

To visually assess the ability of PES to capture cellular heterogeneity and dynamic transitions, nonlinear dimensionality reduction using t-distributed Stochastic Neighbor Embedding (t-SNE) was performed on both GEM of highly variable genes (HVGs) and PES of signaling molecules in the radial progenitor (RD) differentiation dataset. To ensure a fair comparison between expression-based and PES-based representations, the top 10% most HVGs were selected from the GEM, consistent with the PES strategy of using the top 10% signaling molecules. As illustrated in Figures S2A–B, the PES visualizations of signaling genes demonstrate clearer clustering and separation between different time points, where cell populations are more clearly delineated compared to the GEM visualizations of HVGs.

Moreover, to quantitatively compare clustering performance between PES and GEM representations, five clustering algorithms—KMedoids [18], KMeans [19], HDBSCAN [20], DBSCAN [21], and hierarchical clustering [22]—were applied to both representations across the differentiation datasets. Clustering quality was evaluated using the Adjusted Rand Index (ARI), which measures agreement between inferred clusters and reference cell-type annotations while correcting for random assignments. As shown in Figure S2C, the radar plot indicates that the PES representation of signaling genes outperforms the GEM representation of HVGs, as reflected by higher scores in clustering performance metrics. These quantitative results provide strong evidence that the signalling molecules identified by scTIDE provides a robust PES-based representation capable of effectively capturing cellular heterogeneity and dynamic transitions in scRNA-seq data.


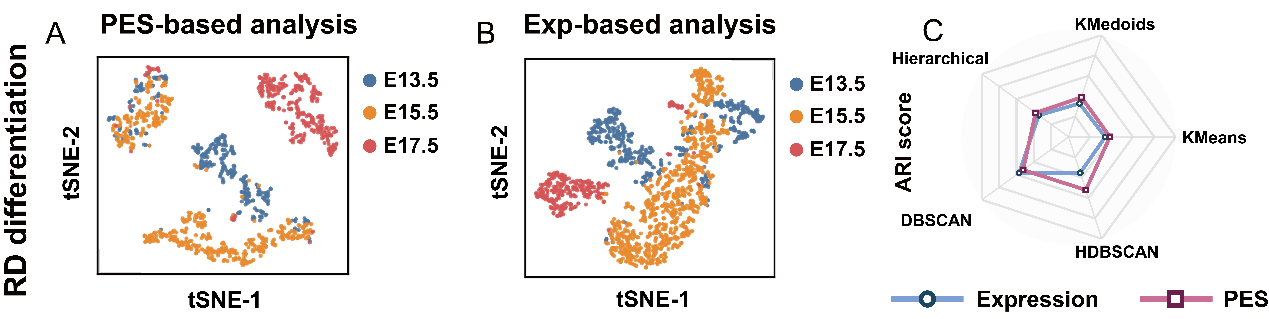
Figure S2. PES-based representation enhances visualization and clustering performance compared with HVG expression. (A) t-SNE visualization based on the PES of signaling molecules, with different colors representing distinct cell types or developmental time points. (B) t-SNE visualization based on the gene expression matrix (GEM) of highly variable genes (HVGs). (C) Radar plots comparing five clustering methods demonstrate that PES-based of signaling molecules representations consistently achieves higher Adjusted Rand Index (ARI) scores than GEM representation of HVGs.

In addition, we performed Uniform Manifold Approximation and Projection (UMAP) analysis to further evaluate the ability of PES-based representations to capture cell-state transitions and cellular heterogeneity. As shown in Figure S3, the UMAP results similarly distinguish cellular states at various time points as effectively as the t-SNE analysis, which demonstrates that PES-based representations can robustly capture cell-state transitions and cellular heterogeneity across different time points, regardless of whether UMAP or t-SNE is used for visualization.


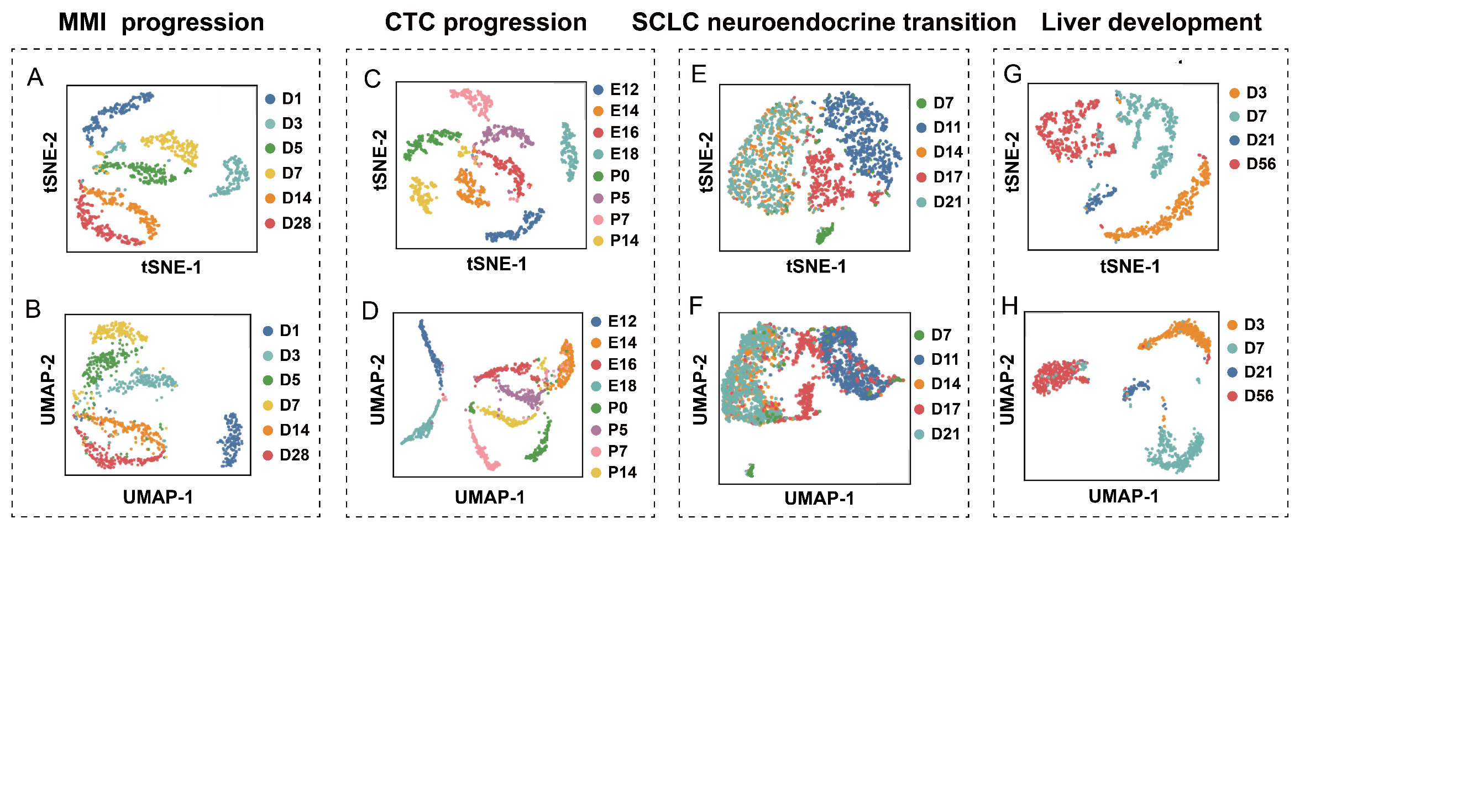
Figure S3. Comparison of PES-based clustering visualized by UMAP and t-SNE. UMAP and t-SNE were applied to visualize PES-based clustering for (A)-(B) MMI progression data, (C)-(D) CTC progression data, (E)-(F) SCLC neuroendocrine transition data, and (G)-(H) liver development data, respectively.

**G. Distributional transport map of signaling molecules**

The distribution map derived from the before-transition state to critical transition state via scTIDE enables us to describe systematic characterization of disease progression and developmental dynamics. At the identified critical stage, a subset of genes, termed signaling molecules (defined as the top 10% of genes with the highest PES values), is selected to investigate their role in governing system transitions. Specifically, in this study, principal component analysis (PCA) [23] was applied to visualize the major transformation patterns in the distribution of signaling molecules across different stages of progression.

As illustrated in Figures S4 A–C, the distributional transport trajectories across three distinct single-cell datasets (including Liver development, SCLC neuroendocrine transition, and MP development) from both disease and developmental systems exhibit a consistent pattern. When the system is far from the critical point, the perturbed distribution remains closely aligned with the reference distribution, whereas a pronounced and abrupt divergence emerges as the system approaches the critical transition. These findings highlight the intrinsic instability of the critical state and underscore the abrupt nature of disease deterioration or developmental shifts. Collectively, these observations demonstrate that scTIDE effectively captures distributional perturbations and robustly identifies critical transitions across diverse disease and developmental contexts.





Figure S4. PCA-based visualization of stage distributional transport from the before-transition state to critical transition state across three single-cell datasets: (A) liver development, (B) SCLC neuroendocrine transition, and (C) MP development.

**H. Functional analysis of signaling molecule**

We performed GO enrichment analyses on the identified signaling genes in the MMI progression, SCLC neuroendocrine transition and liver development datasets. For the MMI progression and SCLC neuroendocrine transition datasets, the identified signaling genes were significantly enriched in biological processes related to actin filament organization and regulation of apoptosis (Figures S5A-B). When applied to the liver development data, the identified signaling genes were predominantly enriched in amino acid metabolism, ATP synthesis coupled electron transport, and generation of precursor metabolites and energy (Figure S5C), highlighting their potential roles in the corresponding developmental processes. In addition, for both liver development and SCLC neuroendocrine transition datasets, several identified signaling genes have been reported in the literature to have important functions in the associated biological processes (Tables S3 and S4), further supporting the functional relevance of these scTIDE-identified signaling molecules.


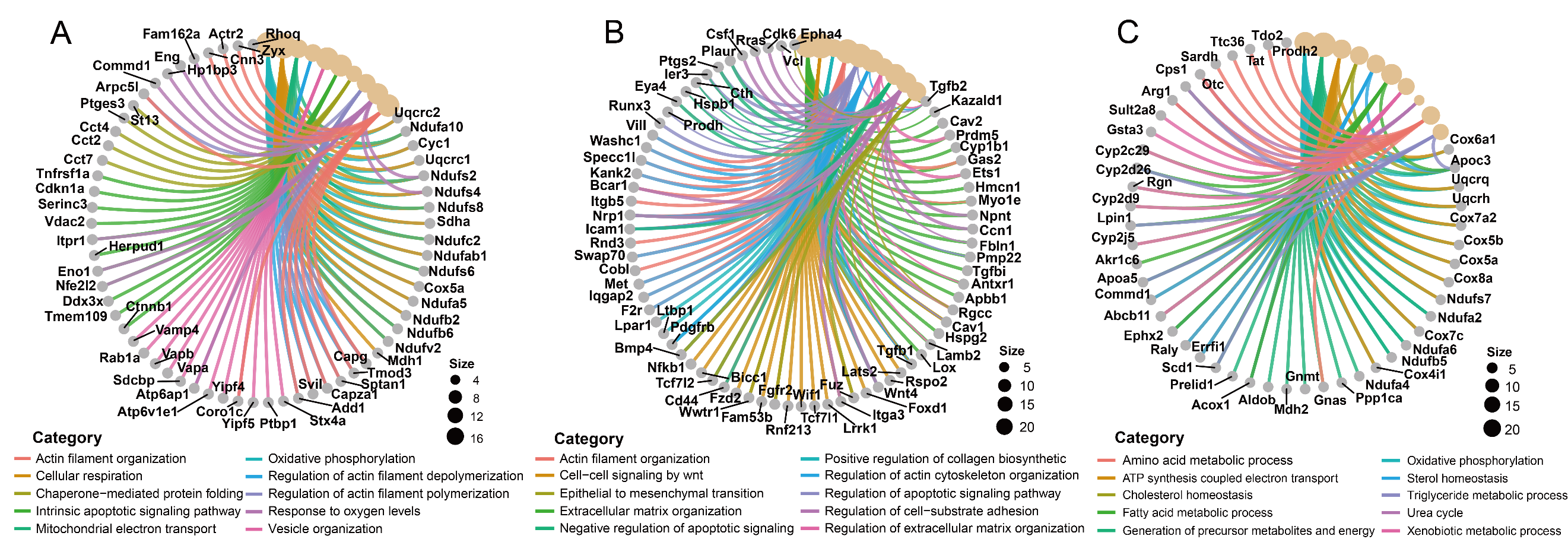


Figure S5. Results GO enrichment analyses of the identified signaling genes in the (A) MMI progression data, (B) SCLC neuroendocrine transition data, and (C) liver development data. The analyses indicate that these signaling genes are functionally associated with the corresponding biological processes.

**Table S3. Information of some key signaling genes in liver development data**

| Gene | Location | Relation with liver development | PMID |
| --- | --- | --- | --- |
| *HNF4A* | Nucleus, Nucleoplasm | *HNF4A* regulates the formation of hepatic progenitor cells from iPSC-derived endoderm by promoting efficient recruitment of RNA polymerase II | 30597922 |
| *ABCB11* | Plasma membrane | *ABCB11* encodes a canalicular bile salt export transporter and is associated with functional maturation of hepatocytes, contributing to bile acid transport and hepatic metabolic homeostasis. | 15791618 |
| *PROX1* | Cytoplasm, Nucleus | *PROX1* serves as a key regulator of embryonic development and organogenesis, including neurogenesis and the development of the heart, lens, liver, pancreas, and lymphatic system | [22733308](https://pubmed.ncbi.nlm.nih.gov/22733308) |
| *CEBPA* | Cytosol | *CEBPA* is a transcription factor essential for coordinating cell differentiation and proliferation arrest in myeloid cells, adipocytes, hepatocytes, and lung tissue | 14660596 |
| *FOXA1* | Nucleus | *FOXA1 and FOXA2* are jointly required for hepatic specification; loss of both factors prevents liver bud formation and abolishes hepatoblast marker expression | 15959514 |
| *FOXA2* | Nucleus | *FOXA2* cooperates with *FOXA1* in the foregut endoderm to drive hepatic specification and liver bud formation | 15959514 |
| *ONECUT1* | Nucleus | *ONECUT1* participates in early liver expansion, and Onecut transcription factors regulate hepatoblast migration during early liver development | 17936262 |
| *HHEX* | Nucleus | *HHEX* is essential for the transition of definitive endoderm into a pseudostratified, cell-emergent epithelium, a key morphogenetic process during early liver bud formation | 16364283 |

**Table S4. Information of some key signaling genes in SCLC neuroendocrine transition data**

| Gene | Location | Relation with SCLC progression | PMID |
| --- | --- | --- | --- |
| *CD44* | Plasma membrane | *CD44* expression increases during SCLC progression and correlates with the acquisition of cancer stem cell-like properties | 33607458 |
| *Met* | Plasma membrane | *c-Met* signaling is involved in SCLC cell proliferation, migration, and invasion, and inhibition of this pathway suppresses malignant phenotypes in H446 SCLC cells | 32682383 |
| *HES1* | Nucleus | *HES1* is a Notch pathway effector whose protein expression has been assessed in SCLC patients and associated with clinical characteristics and prognosis after platinum-based chemotherapy | 33104707 |
| *Cav1* | Caveolae | Higher *Cav1* expression inhibits cisplatin- and UV-induced apoptosis in SCLC cells, suggesting a role in therapy resistance and tumor cell survival | 22545709 |
| *ICAM-1* | Plasma membrane | *ICAM-1* supports SCLC cell adhesion to vascular endothelium, thereby promoting metastatic dissemination and contributing to poor prognosis | 15387368 |
| *REST* | Nucleus | *REST* acts as a tumor suppressor in SCLC, and its loss leads to deregulation of AKT signaling, which is important for SCLC cell proliferation and survival | 20697351 |
| *uPAR* | Plasma membrane | Elevated serum levels of the liberated *uPAR* domain I are independently associated with shorter overall survival in SCLC, suggesting its role in invasion, progression, and prognosis | 23030781 |
| *MDR1* | Plasma membrane | *MDR1*-mediated drug efflux is involved in multidrug resistance in SCLC cell lines, including resistance to drugs such as etoposide | 7834606 |

To further validate the biological relevance of the signaling molecules identified by scTIDE, we performed functional analyses on three embryonic development single-cell datasets (including RD differentiation, hESC differentiation and MP Development). These analyses provide mechanistic insights into the roles of signaling molecules during developmental progression. Specifically, transcription factor (TF) target analysis is conducted to uncover certain signaling genes acting as upstream transcriptional regulators, thereby facilitating the prediction of TFs that may function as key regulators of critical biological processes (Figures S6A–C). Functional enrichment analysis further demonstrates that these signaling molecules are significantly enriched in development-related pathways, supporting their functional involvement in embryonic development (Figures S6D–F). Specifically, in the RD differentiation dataset, signaling molecules show significant enrichment in neurodevelopment-related biological processes, reflecting the progressive maturation of neural stem cells toward committed neural lineages (Figure S6D). Representative pathways, such as neural precursor cell proliferation, neuron fate commitment, and central nervous system neuron differentiation, indicate a developmental transition from progenitor expansion to lineage-specified neuronal differentiation. Additionally, enrichment of gliogenesis and glial cell differentiation highlights the coordinated emergence of glial lineages, underscoring the tightly regulated balance between neurogenesis and gliogenesis during RD-induced neural development [24]. For the hESC differentiation dataset, signaling molecules are significantly enriched in early developmental and germ layer–associated biological processes (Figure S6E). Pathways such as primary germ layer formation and endoderm development indicate activation of early lineage specification programs. In parallel, enrichment of epithelial-to-mesenchymal transition (EMT) and mesenchymal cell differentiation reflects dynamic cell-state remodeling underlying tissue patterning. Notably, activation of the BMP signaling pathway highlights the central role of BMP-mediated signaling in germ layer specification and hESC fate determination during early embryogenesis [25,26]. When applied to MP development dataset, signaling molecules are enriched in pancreatic lineage–specific and metabolic regulatory processes (Figure S6F), reflecting progressive differentiation of pancreatic progenitors toward functional endocrine fates. Representative pathways such as pancreas development, pancreatic A-cell differentiation, and pancreatic B-cell differentiation indicate lineage commitment toward endocrine cell types. Concurrent enrichment of insulin secretion, regulation of insulin secretion, and glucose homeostasis highlights the functional maturation of pancreatic endocrine cells and the establishment of glucose-responsive regulatory programs. Collectively, these pathways delineate a coordinated developmental trajectory linking pancreatic progenitor differentiation with the acquisition of hormone secretion and metabolic control functions [27,28].


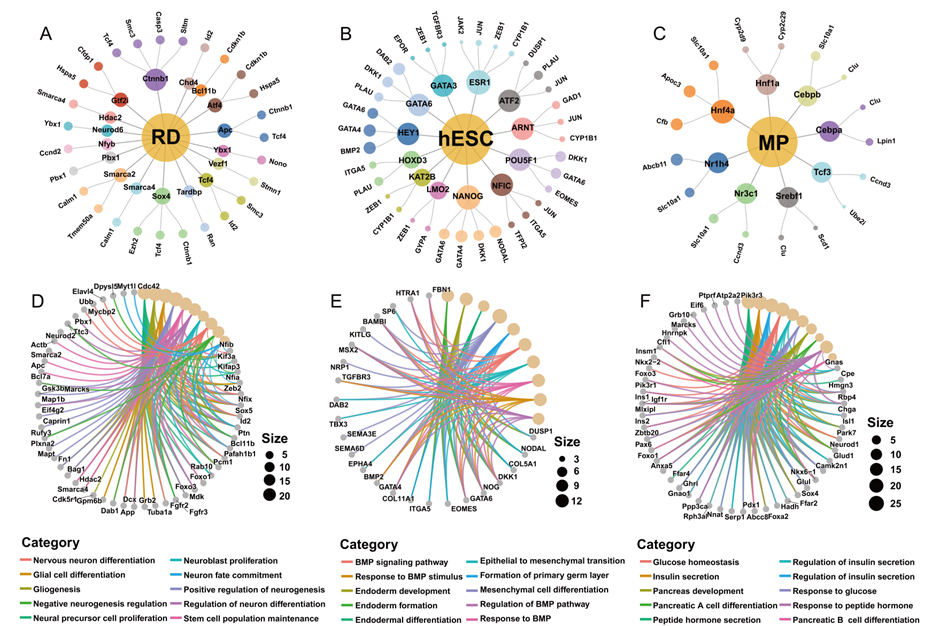
Figure S6. Functional analysis of the signaling molecules implicated in embryonic development. The transcription factor (TF) target analysis is conducted to uncover certain signaling genes acting as upstream transcriptional regulators of downstream molecules associated with key biological progression in three embryonic development single-cell datasets: (A) RD diﬀerentiation, (B) hESC diﬀerentiation, and (C) MP development. The GO pathway enrichment analysis of signaling molecules for these three singe-cell datasets: (D) RD diﬀerentiation, (E) hESC diﬀerentiation, and (F) MP development. The results demonstrate that signaling molecules are mainly enriched in development-related processes.

Besides, to predict whether unknown cells reach a tipping point, a neural network model is trained based on the PES profiles of signaling genes derived from observable cells in the non-critical and critical states. An analogous procedure is applied using expression profiles of the top 10% HVGs. Precision and recall are included to provide a more comprehensive evaluation of classification performance. As shown in Figure S7, prediction models constructed using the PES profiles of signaling genes achieve higher precision and recall than HVG expression-based methods, further supporting the stability of the proposed framework.


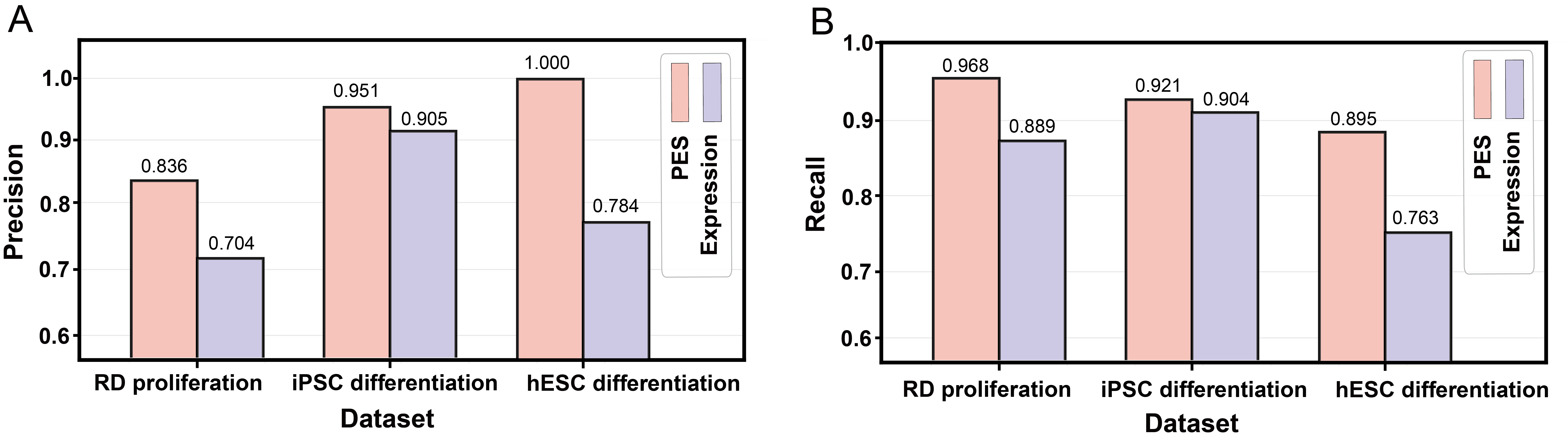


Figure S7. Prediction precision and recall for unseen cells across RD proliferation, iPSC differentiation, and hESC differentiation datasets. From the perspective of precision and recall, the model constructed using PES profiles of signaling genes outperforms HVG expression-based methods.

To elucidate the cell–cell communication mechanisms driving endocrine lineage progression during pancreatic development, we systematically analyzed ligand–receptor–mediated signaling interactions between endocrine progenitors and differentiated cell populations. By integrating inferred intercellular communication probabilities across developmental stages, we aimed to identify key signaling pathways and intermediate cell states that coordinate lineage transitions and functional maturation. This analysis uncovered a prominent signaling network centered on Fev+ cells, emphasizing their regulatory role as an intermediate hub connecting endocrine progenitors with downstream differentiated lineages, as illustrated in Figure S8. Specifically, *Mpzl1–Mpzl1* homophilic interactions mediate direct cell–cell adhesion between Fev+ cells and epsilon cells, facilitating integrin/Src-dependent morphological remodeling and lineage stabilization. Meanwhile, *Mdk–Ptprz1* signaling establishes a paracrine communication axis from Fev+ intermediates to Delta cells, linking Fev+-associated transitional states with downstream endocrine maturation. Together, these findings indicate Fev+ cells as a pivotal regulatory node that integrates structural cell–cell interactions with paracrine growth factor signaling to drive endocrine lineage progression [29-31].





Figure S8. Dot plot of ligand–receptor interactions across different cell types, illustrating that Fev+ cells function as a pivotal regulatory node integrating structural cell–cell interactions with paracrine growth factor signaling to drive endocrine lineage progression.

**I. Robustness analysis of scTIDE with respect to** **the** **kNN parameter** $\boldsymbol{k}$

To examine the influence of the kNN parameter $k$ on scTIDE, we conducted analyses on both simulated data and real SCLC neuroendocrine transition data under different settings of the k-nearest-neighbor (kNN) parameter $k$. For the simulated data, as shown in Figures S9A-C, the PES index consistently identifies the critical state and exhibits similar overall trend patterns across different values of $k$ (5, 10, and 15). A similar pattern is observed in the SCLC neuroendocrine transition data, where the same three values of $k$ were tested (Figures S9D-F). These results indicate that variations in $k$ within a range do not alter the overall behavior of the signal curve, thereby demonstrating the robustness of scTIDE with respect to the parameter $k$.


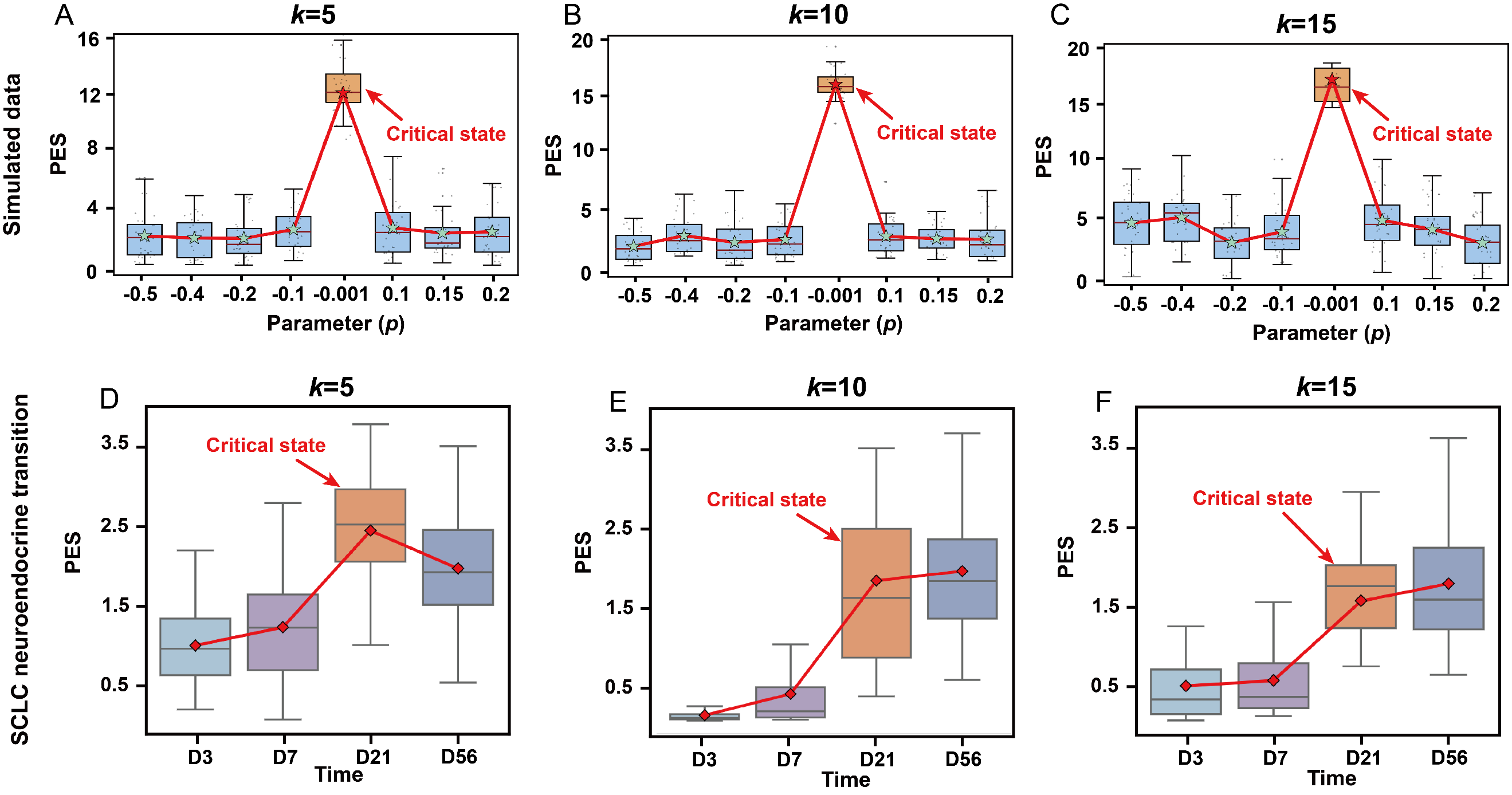


Figure S9. Critical signals observed in simulated and SCLC neuroendocrine transition datasets under different settings of the $k$-nearest-neighbor (kNN) parameter $k$. For the simulated data, (A) $k=$ 5, (B) $k=$ 10, and (C) $k=$ 15, respectively. Similarly, for the SCLC neuroendocrine transition data, (D) $k=$ 5, (E) $k=$ 10, and (F) $k=$ 15, respectively.

**J. Derivation of the** **diffused representation**

We reformulate the personalized diffusion framework and provide a detailed derivation of the diffused representation $D^{*}$, which is obtained by learning node-specific damping factors. Let $M$ denote the diffusion transition matrix of the graph. We consider a personalized diffusion process with node-dependent damping coefficients. The iterative update rule is defined as:

$D^{\left( s \right)}=CD^{\left( s-1 \right)}M+\left( I-C \right)M^{l}$ (S5)

where $D^{\left( s \right)}$ represents the diffusion matrix at iteration $s$, $C$ is a diagonal matrix whose diagonal entry $c_{i}\in[0,1)$ represents the damping factor of node $i$, $I$ is the identity matrix, and $M^{l}$ denotes the restart diffusion matrix obtained by applying the diffusion operator $l$ times. In this study, we set $l=2$ by default.

When the iteration converges as $s$ approaches infinity, the diffusion process can be written as:

$D^{*}=(I-C)(I+CM+C^{2}M^{2}+\cdots+C^{s}M^{s}+\cdots)M^{l}$ (S6)

This Eq. (6) corresponds to a matrix geometric series and defines the closed-form personalized diffusion representation. Each row of $D^{*}$ describes the diffusion distribution of a query node over the graph.

To obtain an analytically tractable form and improve computational efficiency, we consider matrix factorization $M$ *=* $\phi\Lambda\psi^{T},$ where $\Lambda$ is a diagonal matrix whose diagonal entries are the eigenvalues $\lambda_{k},$ and $\phi$ and $\psi$ denote the corresponding left and right eigenvector matrices satisfying $\psi^{T}\phi=I$. Under this decomposition, we obtain the matrix power $M^{s}=\phi\Lambda^{s}\psi^{T}.$ Accordingly, the $(i, j)$-th element of $M^{s}$ admits the following spectral expansion:

$M_{ij}^{(s)}=\sum_{k=1}^{n} \lambda_{k}^{s}\phi_{k}(i)\psi_{k}(j).$ (S7)

Substituting Eq. (S7) into Eq. (S6), we obtain the closed-form expression of the personalized diffusion representation:

${D^{*}}_{ij}=\sum_{k=1}^{n} \frac{(1-c_{i})\lambda_{k}^{l}}{1-c_{i}\lambda_{k}}\phi_{k}(i)\psi_{k}(j).$ (S8)

To simplify notation, we define a matrix $\Sigma$ with entries $\Sigma_{ik}=\frac{(1-c_{i})\lambda_{k}^{l}}{1-c_{i}\lambda_{k}}.$ Using this definition, the the diffused representation admits the compact expression

$D^{*}$ *=* $(\phi\circ\Sigma)\psi^{T},$ (S9)

where $\circ$ denotes the Hadamard (element-wise) product.

To optimize the node-specific damping parameters $c_{i},$ we compute the partial derivative of ${D^{*}}_{ij}$ with respect to $c_{i}$:

$\frac{\partial{D^{*}}_{ij}}{\partial c_{i}}= \sum_{k=1}^{n} \frac{\lambda_{k}^{l}\left( \lambda_{k}-1 \right)}{(1-c_{i}\lambda_{k})^{2}}\phi_{k}\left( i \right)\psi_{k}\left( j \right).$ (S10)

Let $R_{\Sigma}={[\frac{\lambda_{k}^{l}(\lambda_{k}-1)}{\left( 1 - c_{i}\lambda_{k})^{2} \right.}]}_{i\times k} .$ Then the gradient matrix with respect to $C$ can be written compactly as

$\frac{\partial D^{*}}{\partial C}={[\frac{\partial{D^{*}}_{ij}}{\partial c_{i}}]}_{n\times n}=(\phi\circ R_{\Sigma})\psi^{T}.$ (S11)

To determine the optimal damping matrix $C$, we formulate a distribution alignment objective inspired by information-theoretic embedding methods such as t-SNE. Specifically, we minimize the Kullback–Liebler divergence between the ($l+1$)-order diffusion distribution and the personalized diffusion representation:

$L(C)=\mathrm{KL}\text{ }\left( M^{l+1}\parallel D^{*} \right).$ (S12)

This objective enforces the diffused representation $D^{*}$ to approximate the $\left( l + 1 \right)$-order diffusion behavior of the graph while preserving node-level personalization. The optimization is carried out using gradient descent. Starting from an initial damping matrix $C$, the diffused representation $D^{*}$ is computed based on Eq. (S9), the loss $L(C)$ is evaluated using Eq. (S12), and the gradient with respect to $C$ is obtained from Eq. (S11). The procedure is repeated until convergence. After optimization, the learned damping matrix yields the personalized diffusion representation $D^{*}$, which adaptively modulates the spectral response of the diffusion operator and captures node-specific propagation patterns across the graph.

**K. Description of model architecture and training settings in the OT-CFM module**

A detailed description of the neural network architecture and training settings used in the OT-CFM module is provided in Table S5. Specifically, we parameterize the time-dependent velocity field using a fully connected neural network (multilayer perceptron, MLP) with parameters$\theta$, denoted as $v_{\theta}(t,x):(0,1)\times\mathcal{M\to}\mathcal{T}_{\mathcal{M}}$, which is designed to approximate the true velocity field governing the evolution of the probability distribution. The network consists of two hidden layers with 32 neurons each, followed by ReLU activation functions. The model is trained by minimizing the mean squared error between the predicted velocity $v_{\theta}\left( t,x_{t} \right)$ and the conditional velocity $u_{t}\left( x | z \right)$ along transport paths, as defined by the OT-CFM loss $\mathcal{L}_{OT-CFM}$, with a fixed noise scale of $\epsilon$= 0.1, while optimization is performed using the Adam optimizer with a learning rate of $1\times{10}^{-3}$.

**Table RS5. Summary of symbol, model architecture, and training setting in OT-CFM module**

| **Group** | **Item** | **Description** |
| --- | --- | --- |
| Space | $\mathcal{M}$ | Manifold-based space where transport dynamics are modeled |
|  | $\mathcal{T}_{\mathcal{M}}$ | Space of time-dependent velocity fields on $\mathcal{M}$ |
| Variable | $z$ | Paired samples $z=(x_{0},x_{1})$ drawn from the reference and perturbed states, respectively |
|  | $t$ | Continuous time variable along the transport path |
|  | $x_{t}$ | Intermediate state along transport path at time $t$ |
| Velocity  field | $u_{t}(x\vert z)$ | Conditional velocity at position $x$ and time $t$, conditioned on  paired samples $z$ |
|  | $v_{\theta}\left( t,x \right): (0,1)\times\mathcal{M\to}\mathcal{T}_{\mathcal{M}}$ | Function mapping of the parameterized velocity field with parameters $\theta$ that approximates the true velocity field |
| Model parameter | $\theta$ | Trainable parameters of the model |
|  | $\epsilon$ | Hyperparameter controlling the noise scale in OT-CFM sampling, with a value set to 0.1 |
| Model architecture | Hidden layer | 2 fully connected layers |
|  | Hidden unit | 32 neurons per layer |
|  | Activation function | ReLU |
| Training objective/Loss | $\mathcal{L}_{OT-CFM}$ | Minimizing the mean squared deviation between $v_{\theta}\left( t,x_{t} \right)$ and $u_{t}\left( x \vert z \right)$ |
| Optimization setting | Optimizer | Adam optimizer for parameter optimization |
|  | Learning rate | A learning rate of $1\times{10}^{-3}$ used for optimization |

**L. Performance of scTIDE under varying parameter**$\boldsymbol{L}$

We performed the analysis on the SCLC neuroendocrine transition and liver development datasets under varying parameter $L$, ranging from the top 5% to 15% of local graphs ranked by local PES. As presented in Figure S10, similar PES patterns were observed across all tested settings, and the identified critical-transition states remained unchanged. In particular, sharp increases in PES were consistently detected across different threshold choices. Therefore, these results demonstrate that the performance of scTIDE is not strongly dependent on the default setting of the top 10% local graphs and remains stable across a reasonable range of threshold values (typically from the top 5% to 15%).


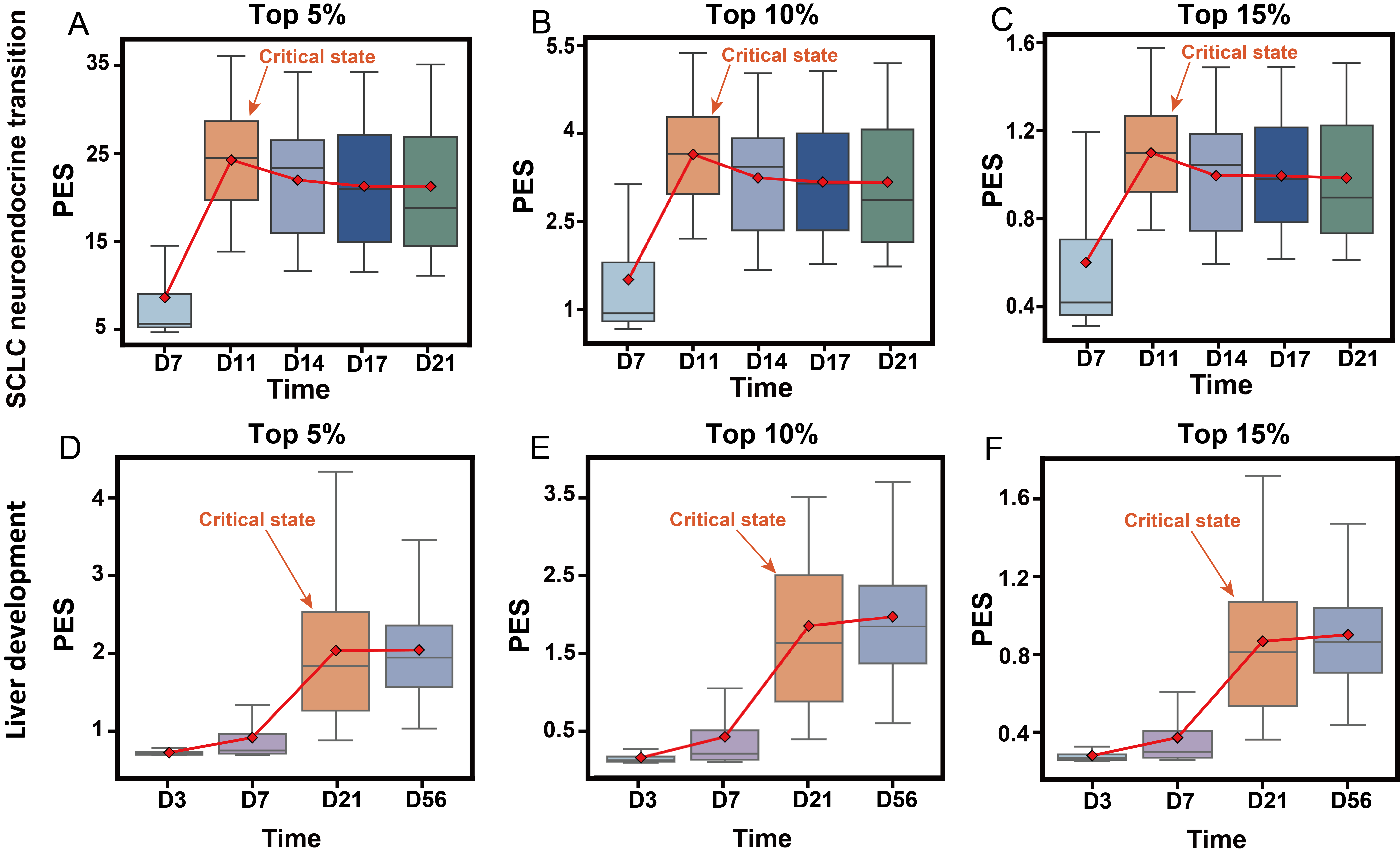
 Figure S10. Robustness analysis of scTIDE with respect to the threshold used for calculating cell-specific PES. For the SCLC neuroendocrine transition data, results obtained using the (A) top 5%, (B) top 10%, and (C) top 15% local graphs with the highest local PES values are shown. For the liver development data, results obtained using the (D) top 5%, (E) top 10%, and (F) top 15% local graphs with the highest local PES values are presented. Similar PES patterns and consistent identification of critical transition states are observed across different threshold settings.

**M.** **Effect size quantification of critical states using Cohen’s d (CSD)**

It is important to note that statistical significance alone is insufficient for characterizing abrupt transitions in complex biological systems. While P-values indicate the probability of observing a difference under the null hypothesis, they provide no information about the strength or biological impact of that difference.

To capture both statistical significance and effect size, we incorporate Cohen’s d (CSD) alongside the P-value metric, which allows the proposed framework to evaluate not only whether a transition is statistically detectable, but also whether it is substantial in magnitude, thereby improving the robustness of tipping-point identification.

Cohen’s d (CSD) is introduced as a complementary metric that provides a standardized and scale-independent measure of critical transition strength, enabling a more objective quantification of how abrupt and biologically meaningful the change in PES is. Specifically, the two-sample t-statistic CSD is used to assess how strongly the mean of an $n₁$-dimensional vector $\hat{X_{1}}$ $=(x_{1}^{1},x_{1}^{2},\ldots,x_{1}^{n_{1}})$ differs from the mean of an $n₂$-dimensional vector $\hat{X_{2}}=(x_{2}^{1},x_{2}^{2},\ldots,x_{2}^{n_{2}})$ relative to their pooled variability, thereby providing a direct measure of the magnitude of change between the two states, that is:

$CSD=\frac{\mathrm{mean}(\hat{X_{1}})-\mathrm{mean}(\hat{X_{2}})}{s_{\text{pooled}}}$ (S13)

where $\mathrm{mean}(\hat{X_{1}})$ and $\mathrm{mean}(\hat{X_{2}})$ are the means of vectors $\hat{X_{1}}$ and $\hat{X_{2}}$ respectively, and $s_{\text{pooled}\text{ }}$ is the pooled standard deviation defined as:

$s_{\text{pooled}}=\sqrt{\frac{(n_{1}-1)\mathrm{Var}(\hat{X_{1}})+(n_{2}-1)\mathrm{Var}(\hat{X_{2}})}{n_{1}+n_{2}-2}}$ (S14)

Here, $Var(\hat{X_{1}})$ and $Var(\hat{X_{2}})$ represent the sample variances of $\hat{X_{1}}$ and $\hat{X_{2}}$ respectively, and $n_{1}$ and $n_{2}$ are their respective sample sizes.

The effect size CSD reflects the practical significance of the difference in PES between the critical state and the before-transition state. Following conventional interpretation guidelines: (1) $\mid CSD\mid<0.2$: negligible effect; (2) $0.2\leq\mid CSD\mid<0.5$: small effect; (3) $0.5\leq\mid CSD\mid<0.8$: medium effect; (4) $\mid CSD\mid\geq0.8$: large effect. Compared with the previous time point $t-1$, a positive and large CSD at time point $t$, signals a pronounced escalation in PES, which reflects a meaningful amplification of system instability.

**N.** **Description of the eight real single-cell datasets**

To demonstrate the capabilities of the scTIDE method, it has been utilized on eight diverse real-world single-cell datasets, covering disease progression-related contexts such as mouse myocardial infarction (MMI) (EMBL: E-MTAB-7895) obtained from European Molecular Biology Laboratory (https://www.embl.org), cerebellar tumor cells (CTC) progression (GEO: GSE118068), and neuroendocrine transitions in small cell lung cancer (SCLC) (GEO: GSE149179), as well as developmental processes including murine pancreatic (MP) development (GEO: GSE1322188), liver development (GEO: GSE171993), human embryonic stem cell (hESC) differentiation (GEO: GSE75748), induced pluripotent stem cell (iPSC) differentiation (PMCID: PMC5338498), and radial progenitor (RD) proliferation(GEO:GSE107122) sourced from the Gene Expression Omnibus database (http://www.ncbi.nlm.nih.gov/geo).

The MMI dataset comprises live, nucleated interstitial cells profiled at homeostasis (7467 cells) and at Day 1 (5069 cells), Day 3 (5399 cells) (inflammatory phase), and Day 5 (6673 cells), Day 7 (6991 cells) (proliferative phase), as well as Day 14 (9574 cells), Day 28 (8413 cells) (maturation phase of post-MI) from five mice. This dataset captures the critical transition from inflammatory injury response to early myofibroblast activation during the high-risk cardiac rupture window (Days 3-4), followed by gradual restoration toward homeostatic cell-type composition during the maturation phase [5]. Gene expression profiling data can be accessed at: https://www.ebi.ac.uk/biostudies/arrayexpress/studies/E-MTAB-7895.

The CTC progression dataset captures neural lineage development across late embryonic to postnatal stages: E10 (7742 cells), E12 (7540 cells), E14 (6068 cells), E16 (7383 cells), E18 (5950 cells), P0 (4809 cells), P5 (11707 cells), P7 (7179 cells), and P14 (4515 cells) [6]. The gene expression profiling data can be download at: https://www.ncbi.nlm.nih.gov/geo/query/acc.cgi?acc=GSE118068.

The SCLC dataset characterizes MYC-driven small cell lung cancer progression across a temporal series, capturing the transition from ASCL1-high neuroendocrine states (Days 4-7) to predominantly non-neuroendocrine phenotypes (Days 11-21) in both cultured and in vivo tumor contexts. The cells can be divided into several time points: Day 1 (1069 cells), Day 7 (119 cells), Day 11 (1165 cells), Day 14 (520 cells), Day 17 (470 cells), and Day 21 (691cells) [7]. Gene expression profiling data can be retrieved from: https://www.ncbi.nlm.nih.gov/geo/query/acc.cgi?acc=GSE149179.

The MP development dataset comprises scRNA-seq profiles of the E15.5 murine pancreatic development, which describes differentiation from low-Neurog3 (673 cells) and high-Neurog3 (642 cells) endocrine progenitors through a transitional Fev⁺ intermediate state (592 cells) toward multiple endocrine lineages, including the generation of alpha, beta, delta, and epsilon cell fates [8]. Gene expression profiling data can be available at: https://www.ncbi.nlm.nih.gov/geo/query/ acc.cgi?acc= GSE132188.

The liver development dataset comprises scRNA-seq profiles of mouse liver cells collected at postnatal Day 1 (214 cells), Day 3 (384 cells), Day 7 (425 cells), Day 21 (102 cells), and Day 56 (433 cells), spanning neonatal hepatocytes (D1–D7), a transitional stage (D21), and mature adult hepatocytes (D56) to characterize postnatal liver development [9]. Gene expression profiling data can be accessed at: https://www.ncbi.nlm.nih.gov/geo/query/acc.cgi?acc=GSE171993.

The hESC differentiation dataset characterizes the trajectory from pluripotent human embryonic stem cells (hESC) through a mesendodermal intermediate to definitive endoderm cells (DEC). A total of 758 single cells were profiled across six time points: 0 h (92 cells), 12 h (102 cells), 24 h (66 cells), 36 h (172 cells), 72 h (138 cells), and 96 h (188 cells) [10]. Gene expression profiling data are deposited at: https://www.ncbi.nlm.nih.gov/geo/query/ acc.cgi?acc=GSE75748.

The iPSC differentiation dataset comprises single-cell transcriptomic profiles collected at eight distinct time points: Day 0 (231 cells), Day 1 (136 cells), Day 1.5 (59 cells), Day 2 (181 cells), Day 2.5 (98 cells), Day 3 (226 cells), Day 4 (434 cells), and Day 5 (327 cells) [11]. Gene expression profiling data can be obtained at: https://pubmed.ncbi.nlm.nih.gov/28167799/.

The RD proliferation dataset characterizes radial progenitor transcriptional dynamics from E11.5 to E17.5. Single cells were profiled at four developmental stages: E11.5 (1,418 cells), E13.5 (1,137 cells), E15.5 (2,955 cells), and E17.5 (880 cells) [12]. The dataset illustrates maintenance of progenitor identity during the shift from proliferative to non-proliferative states. Gene expression profiling data can be accessed at: https://www.ncbi.nlm.nih.gov/geo/query/acc.cgi?acc=GSE107122.

**O. Stability analysis of scTIDE with respect to the number of HVGs**

To evaluate whether PES calculation is sensitive to the number of highly variable genes (HVGs) selected for analysis, we performed further sensitivity analyses on both the SCLC neuroendocrine transition and liver development datasets by varying the number of selected HVGs, including 1500, 2000, 2500, and 3000 genes. It can be seen from Figure S11 that the resulting PES curves remain consistent across different HVGs numbers, with stable identification of the critical transitions. These results indicate that the proposed scTIDE is not sensitive to moderate changes in the number of HVGs, thereby supporting the stability of the framework in terms of HVG selection.


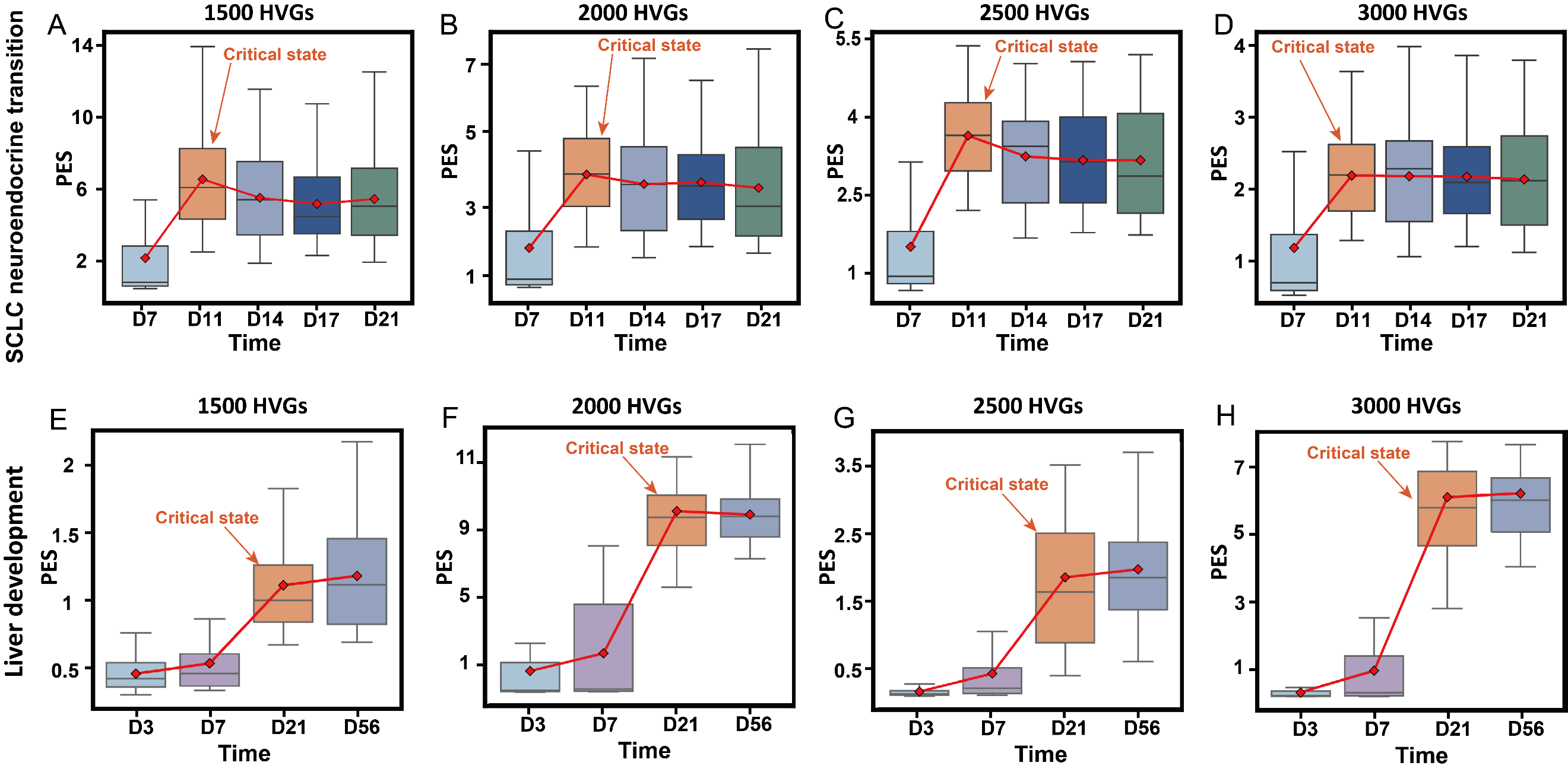


Figure S11. Critical-transition signals detected by scTIDE under different the number of HVGs selection thresholds in the SCLC neuroendocrine transition and liver development datasets. For the SCLC neuroendocrine transition data, (A) 1500 HVGs, (B) 2000 HVGs, (C) 2500 HVGs, and (D) 3000 HVGs, respectively. Similarly, for the liver development data, (E) 1500 HVGs, (F) 2000 HVGs, (G) 2500 HVGs, and (H) 3000 HVGs, respectively.

**References**

[1] Chen L, Liu R, Liu ZP, Li M, Aihara K. Detecting early-warning signals for sudden deterioration of complex diseases by dynamical network biomarkers. Sci Rep. 2012;2:342.

[2] Liu R, Chen P, Aihara K, Chen L. Identifying early-warning signals of critical transitions with strong noise by dynamical network markers. Scientific reports. 2015;5.

[3] Ronen M, Rosenberg R, Alon U *et al*. Assigning numbers to the arrows: parameterizing a gene regulation network by using accurate ex-pression kinetics. *Proc Natl Acad Sci USA* 2002; **99**: 10555–60.

[4] Khanin R, Vinciotti V, Mersinias V *et al*. Statistical reconstruction of transcription factor activity using Michaelis–Menten kinetics. *Biometrics* 2007; **63**: 816–23.

[5] Forte E, Skelly D A, Chen M, et al. Dynamic interstitial cell response during myocardial infarction predicts resilience to rupture in genetically diverse mice[J]. Cell reports, 2020, 30(9): 3149-3163. e6.

[6] Tran T N, Bader G D. Tempora: cell trajectory inference using time-series single-cell RNA sequencing data[J]. PLoS computational biology, 2020, 16(9): e1008205.

[7] Ireland A S, Micinski A M, Kastner D W, et al. MYC drives temporal evolution of small cell lung cancer subtypes by reprogramming neuroendocrine fate[J]. Cancer cell, 2020, 38(1): 60-78. e12.

[8] Lange M, Bergen V, Klein M, et al. CellRank for directed single-cell fate mapping[J]. Nature methods, 2022, 19(2): 159-170.

[9] Liang Y, Kaneko K, Xin B, et al. Temporal analyses of postnatal liver development and maturation by single-cell transcriptomics[J]. Developmental cell, 2022, 57(3): 398-414. e5.

[10] Chu LF, Leng N, Zhang J, Hou Z, Mamott D, Vereide DT, Choi J, Kendziorski C, Stewart R, Thomson JA. Single-cell RNA-seq reveals novel regulators of human embryonic stem cell differentiation to definitive endoderm. Genome Biol. 2016 Aug 17;17(1):173.

[11] Bargaje R, Trachana K, Shelton M N, et al. Cell population structure prior to bifurcation predicts efficiency of directed differentiation in human induced pluripotent cells[J]. Proceedings of the National Academy of Sciences, 2017, 114(9): 2271-2276.

[12] Yuzwa S A, Borrett M J, Innes B T, et al. Developmental emergence of adult neural stem cells as revealed by single-cell transcriptional profiling[J]. Cell reports, 2017, 21(13): 3970-3986.

[13] Peng X, Qiao R, Li P, Chen L. DNFE: Directed network flow entropy for detecting tipping points during biological processes. PLoS Comput Biol. 2025 Jul 29;21(7):e1013336.

[14] Liu R, Chen P, Chen L. Single-sample landscape entropy reveals the imminent phase transition during disease progression. Bioinformatics. 2020 Mar 1;36(5):1522-1532. doi: 10.1093/bioinformatics/btz758. Erratum in: Bioinformatics. 2020 Apr 15;36(8):2644.

[15] Hua W, Cui R, Yang H, Zhang J, Liu C, Sun J. Uncovering critical transitions and molecule mechanisms in disease progressions using Gaussian graphical optimal transport. Commun Biol. 2025 Apr 6;8(1):575.

[16] Li L, Xu Y, Yan L, Li X, Li F, Liu Z, Zhang C, Lou Y, Gao D, Cheng X, Chen L. Dynamic network biomarker factors orchestrate cell-fate determination at tipping points during hESC differentiation. Innovation (Camb). 2022 Dec 20;4(1):100364.

[17] Zhong J, Li J, Gu X, Ding D, Ling F, Chen P, Liu R. sPGGM: a sample-perturbed Gaussian graphical model for identifying pre-disease stages and signaling molecules of disease progression. Natl Sci Rev. 2025 May 14;12(8):nwaf189.

[18] Ciortan M, Defrance M. Contrastive self-supervised clustering of scRNA-seq data[J]. BMC bioinformatics, 2021, 22(1): 280.

[19] Baker D N, Dyjack N, Braverman V, et al. Fast and memory-efficient scRNA-seq k-means clustering with various distances[C]//Proceedings of the 12th ACM International Conference on Bioinformatics, Computational Biology, and Health Informatics. 2021: 1-8.

[20] Song J, Liu Y, Zhang X, et al. Entropy subspace separation-based clustering for noise reduction (ENCORE) of scRNA-seq data[J]. Nucleic acids research, 2021, 49(3): e18-e18.

[21] Feng C, Liu S, Zhang H, et al. Dimension reduction and clustering models for single-cell RNA sequencing data: a comparative study[J]. International journal of molecular sciences, 2020, 21(6): 2181.

[22] Žurauskienė J, Yau C. pcaReduce: hierarchical clustering of single cell transcriptional profiles[J]. BMC bioinformatics, 2016, 17(1): 140.

[23] Reich D, Price A L, Patterson N. Principal component analysis of genetic data[J]. Nature genetics, 2008, 40(5): 491-492.

[24] Gouti M, Tsakiridis A, Wymeersch F J, et al. In vitro generation of neuromesodermal progenitors reveals distinct roles for wnt signalling in the specification of spinal cord and paraxial mesoderm identity[J]. PLoS biology, 2014, 12(8): e1001937.

[25] Murry C E, Keller G. Differentiation of embryonic stem cells to clinically relevant populations: lessons from embryonic development[J]. Cell, 2008, 132(4): 661-680.

[26] Pera M F, Andrade J, Houssami S, et al. Regulation of human embryonic stem cell differentiation by BMP-2 and its antagonist noggin[J]. Journal of cell science, 2004, 117(7): 1269-1280.

[27] Pan F C, Wright C. Pancreas organogenesis: from bud to plexus to gland[J]. Developmental dynamics, 2011, 240(3): 530-565.

[28] Bastidas-Ponce A, Tritschler S, Dony L, et al. Comprehensive single cell mRNA profiling reveals a detailed roadmap for pancreatic endocrinogenesis[J]. Development, 2019, 146(12): dev173849.

[29] Byrnes L E, Wong D M, Subramaniam M, et al. Lineage dynamics of murine pancreatic development at single-cell resolution[J]. Nature communications, 2018, 9(1): 3922.

[30] Bastidas-Ponce A, Tritschler S, Dony L, et al. Comprehensive single cell mRNA profiling reveals a detailed roadmap for pancreatic endocrinogenesis[J]. Development, 2019, 146(12): dev173849.

[31] Fujikawa A, Noda Y, Yamamoto H, et al. Mice deficient in protein tyrosine phosphatase receptor type Z (PTPRZ) show reduced responsivity to methamphetamine despite an enhanced response to novelty[J]. PloS one, 2019, 14(8): e0221205.
